# Seed endophytes of malting barley from different locations are shaped differently and are associated with malt quality traits

**DOI:** 10.1186/s12870-025-06089-6

**Published:** 2025-02-05

**Authors:** Oyeyemi Ajayi, Ramamurthy Mahalingam

**Affiliations:** USDA-ARS, Cereal Crops Research Unit, 502 Walnut Street, Madison, WI 53726 USA

**Keywords:** Barley, Endophytes, Malt quality, Microbiome, Seeds

## Abstract

Maximizing microbial functions for improving crop performance requires better understanding of the important drivers of plant-associated microbiomes. However, it remains unclear the forces that shapes microbial structure and assembly, and how plant seed-microbiome interactions impact grain quality. In this work, we characterized the seed endophytic microbial communities of malting barley from different geographical locations and investigated associations between microbial (bacterial and fungal) species diversity and malt quality traits. Host genotype, location, and interactions (genotype x location) significantly impacted the seed endophytic microbial communities. Taxonomic composition analysis identified the most abundant genera for bacterial and fungal communities to be *Bacillus* (belonging to phylum Firmicutes) and *Blumeria* (belonging to phylum Ascomycota), respectively. We observed that a greater proportion of bacterial amplicon sequence variants (bacterial ASVs) were shared across genotypes and across locations while the greater proportion of the fungal ASVs were unique to each genotype and location. Association analysis showed a significant negative correlation between bacterial alpha diversity indices (Faith PD and Shannon indices) and malt quality traits for barley protein (BP), free amino nitrogen (FAN), diastatic power (DP) and alpha amylase (AA), while fungal alpha diversity (Shannon and Simpson) showed significant negative relationship with β-D-glucan content. In addition, some bacterial and fungal genera were significantly associated with malt extract (ME) -a key trait for maltsters and brewers. We conclude that barley genotype, location, and their interactions shape the seed endophytic microbiome and is key to microbiome manipulation and management during barley production and/or malting.

**Supplementary Information:**

The online version contains supplementary material available at 10.1186/s12870-025-06089-6.

## Introduction

Substantial efforts geared towards characterizing the underlying processes driving host-microbe interactions have demonstrated the indisputable role of microorganisms to agroecosystem health [37]. The specialized symbiotic relationships between microbes and their host plants are due to their interdependence for nutrient supply [34], and have enabled host organisms to live in niche habitats. The microbial communities associated with plants, commonly referred to as the plant microbiome, are essential to plant growth and resistance to phytopathogens, and the seed associated microbes are co-opted early on at the seed stage to influence seedling growth vigor and plant agroecosystem adaptation [35]. With seed endophytes (microbes colonizing internal seed tissues) containing specialized microorganisms driven by the host recruitment, microbiome-related traits have recently become an attractive target for breeders due to their contributions to plant health, stress tolerance and productivity. Seed endophytes like bacteria can metabolize nutrients for plant uptake (e.g. nitrogen-fixation, phosphate solubilization and siderophore production for iron uptake) and improve tolerance to abiotic and biotic stress or produce phytohormones (e.g. auxins) for plant growth and fitness [30, 33].

Barley (*Hordeum vulgare*) is a resilient crop that is widely distributed worldwide and is cultivated mostly in highly productive agricultural systems for food, feed, fuel, and alcohol production [81]. ND Genesis, AAC Synergy and Conlon are hulled two-row spring malting barley varieties used as “malting check cultivars” by barley breeders to evaluate the malting quality of new barley selections intended for use as malting barley varieties [5]. ND Genesis (western two-rowed varieties and midwestern six-row varieties cross) and Conlon (Bowman*2/Brigitta mutant//ND10232 cross) were developed by North Dakota State University and exhibit moderate resistance to net blotch but are moderately susceptible to Fusarium head blight (FHB) [78, 79]. Similarly, AAC Synergy ( TR02267/Newdale cross), developed at the Agriculture and Agri-Food Canada (AAFC) Brandon Research Centre, exhibits moderate resistance to both net blotch and spot blotch, while also showing moderate susceptibility to FHB and loose smut [62]. The biodiversity of barley germplasm has led to the development of highly productive modern cultivars but also resulted in significant compositional shifts in their microbiomes [88, 89]. Yield losses due to sub-optimal cultivar performance have partly been ascribed to the significant reduction in genetic diversity of plant beneficial microbes [31]. While there may likely be an enrichment of opportunistic phytopathogens in cultivars with microbial dysbiosis, evidence highlighted the role of the host immune system in promoting microbiota homeostasis and mitigating potential virulent effects on opportunistic pathogens [63, 87].

In the field, diverse bacteria and fungi present on the barley may have originated either by vertical (acquired directly from the parent) or horizontal (acquired from the surrounding environment) transmission. In addition to barley seed carrying forward the progeny which germinates into a new plant [80], they are important starting raw material in malting and brewing industry. Following harvest, microbes continue to interact with the living grain prior to malting to overcome dormancy [18] and the nature of their interactions could determine malt quality outcomes. For example, *Bacillus* spp. abundance can cause excessive acidification and nitrosamine formation by reducing nitrate to nitrite during wort production [100], while *Fusarium* spp.*,* a fungal pathogen secretes mycotoxins that survive the brewing process and can be detected in finished beer [39, 58]. Besides producing mycotoxins such as deoxynivalenol, *Fusarium* spp. are known to reduce wort β-D-glucan and viscosity and increase soluble nitrogen, free amino nitrogen, and wort color compared to wort produced from control malt, largely due to the action of fungal enzymes [82, 98]. An infection of cereals with *Fusarium* spp. can also contribute to starch degradation [98]. One significant challenge, even with an understanding of these effects, is the difficulty in predicting when *Fusarium*-related malt quality defects will occur [50], especially when variability among strains of *F. graminearum* have differential impacts on malt quality [65, 73]. Additionally, the genera *Bipolaris*, *Pyrenophora*, *Phaeosphaeria*, *Alternaria*, and *Fusarium* are prevalent in barley grain; the latter two genera, in particular, are well known mycotoxin producers in barley [26, 48].

Malting processes especially during the germination stage has been demonstrated to promote microbial activity [18]. Depending on their quantity, type and metabolites produced these microbes can have deleterious or beneficial consequences for malt quality [60]. Perturbance in wort separation has been reported due to non-viable malt derived bacteria (Walker et al., 1997). Species of *Bacillus, Microccocus, Erwinia and Pantoea agglomerans*, discharge exopolysaccharides from the grain matrix with debilitating effects on wort filtration performance [60]. Certain fungal species are more deleterious in their effect on malt quality, causing process failures such as premature yeast flocculation during beer fermentation [54]. Conversely, some can be beneficial to malt quality; for instance, the antagonistic yeast *Wickerhamomyces anomalus* can be used as a malting starter culture to improve malt quality [61].

Although our knowledge of seed endophytes as beneficial microbes critical for plant growth and tolerance to biotic and abiotic stress are constantly increasing, gaps exist in our understanding of the microbial structure and diversity of the malting barley seed microbiome and their resultant effects on malt quality. To address this fundamental knowledge gap, we characterized the malting barley seed endophytic microbiomes and investigated their associations with malt quality traits. We focused on seed endophytes, recognizing that the steeping process in malting involves the continuous removal of effluent water, which contains microbial contaminants from barley seed surface and is consistently eliminated during the steeping stage [104]. Consequently, we believe that seed endophytes play a vital role in the subsequent stages of the malting process.

Growth of indigenous microbiota have a huge influence on the malt quality and wort filtration performance [18, 94]. This prompted us to examine the effects of genotype (ND Genesis, Conlon and AAC Synergy) and location (St. Paul, MN; Crookston, MN; Casselton, ND and Carrington, ND) on the malting barley seed endophytic bacteria composition and assembly. We also set to investigate associations between seed microbial taxa/diversity and malt quality traits. We answered these questions by using next generation sequencing (16S rRNA and ITS gene sequencing) to characterize both bacterial and fungal communities, while associating bacterial diversity and abundance with malt quality.

## Materials and methods

### Barley seed germplasm

Visually clean seeds from the 2022 crop year were plump tested using a sizing tray having 6/64″ slits. Seeds of Conlon, AAC Synergy and ND Genesis that were retained on the sieve during shaking were utilized in this study. These malting cultivars are well adapted to all areas of North Dakota and adjacent parts of Minnesota, Montana, and South Dakota where barley is grown [78]. To this end, seeds of each malting barley cultivars were grown under field conditions in Minnesota (St Paul and Crookston) and North Dakota (Carrington and Casselton). They were pooled together to form a composite and later subsampled into two parts: the first part was set aside for bacterial and fungal endophytic characterization and the second part was set aside for micro malting. The daily average temperatures and the amount of rainfall for the field locations [110] were collected for the months of April through August (Supplementary Table S11).

### Surface sterilization of seed samples

One gram of barley seeds of the three malting barley varieties grown across four locations was taken in quadruplicate to give a total of 48 samples (3 cultivars X 4 locations X 4 replicates = 48 samples total) for the isolation of genomic DNA. Seed surface sterilization was carried out following a method described previously [7]. Specifically, barley seeds were first transferred to sterile 50 ml tubes and rinsed with sterile distilled water and washed with 70% ethanol for 3 min, followed by treatment with 1% sodium hypochlorite for 150 s. After sodium hypochlorite sterilization, each replicated sample was further subjected to another 70% ethanol wash and subsequently rinsed five times with sterile distilled water. To evaluate the efficiency of the sterilization, 100 µl of the water from the last rinse from each sample was plated on nutrient agar and tryptic soy agar plates and incubated at 30 °C for 3 days. Sterilized seeds were subsequently dried in an oven at 50 °C overnight to remove moisture and subsequently placed in minus 80 °C until further analysis.

### Malt quality analyses

Malt quality analysis for each genotype-location samples were performed following methods described elsewhere [4]. Briefly, one hundred and ten (110) grams (on a dry basis) of each barley sample were malted at the Cereal Crops Research Unit’s Malt Quality Lab (Madison, WI) to estimate malt quality parameters; diastatic power (DP), alpha amylase (AA), malt extract (ME), wort protein (WP), soluble to total protein (ST) ratio, and free amino nitrogen (FAN). The malting procedure consisted of the imbibition of barley grains (i.e., steeping) in a tank that was periodically submerged over a 36-h period to achieve a target moisture content of 45%. Following steeping, the imbibed barley seeds were transferred to germinators and incubated under controlled temperature (16^∘^C), humidity (95%), and airflow conditions for five days. Samples were then subjected to kilning to arrest germination, preserve the modified seed, and bring the moisture levels to about 4%. After the rootlets were removed, malts were ground and subsequently analyzed for malt quality parameters following established procedures approved by the American Society of Brewing Chemists [68].

### DNA isolation for metagenomic sequencing

For total genomic DNA, sterilized samples were grounded with liquid nitrogen and powdered seeds were processed using DNeasy Plant Mini kit (Qiagen) for genomic DNA isolation according to the manufacturer’s protocol. Following extraction, DNA quality was assessed using 0.8% agarose gel, and the purity was determined using a NanoDrop spectrophotometer (Thermo Scientific, USA). Isolated DNA was stored at −80 °C for 16 s rRNA and ITS amplicon sequencing.

### Library preparation and Illumina 16 s and ITS sequencing

The metagenomic libraries (V3-V4 16 s) were constructed and sequenced at the University of Wisconsin-Madison Biotechnology Center using the purified genomic DNA. Qubit® dsDNA HS Assay Kit or Quant-iT™ PicoGreen® dsDNA Assay Kit (ThermoFisher Scientific, Waltham, MA, USA) was used to determine DNA concentration. Illumina’s 16 s Metagenomic Sequencing Library Preparation Protocol, Part #15,044,223 Rev. B (Illumina Inc., San Diego, California, USA) was used for sample preparation with some modifications. The modifications included the sequence of the 16S rRNA gene V3/V4 variable region that was amplified with fusion primers (forward primer 341f: 5’-ACACTCTTTCCCTACACGACGCTCTTCCGATCT(N)_0/6_CCTACGGGNGGCWGCAG−3’, reverse primer 805r: 5’-GTGACTGGAGTTCAGACGTGTGCTCTTCCGATCT(N)_0/6_GACTACHVGGGTATCTAATCC−3’). Region specific primers are indicated by underline and were previously described ( [57]. The ((N)_0/6_) indicates the part of the primer sequences that were modified to add zero or six random nucleotides. Reaction products were then cleaned using AxyPrep Mag PCR clean-up beads (Axygen Biosciences, Union City, CA). This was followed by PCR amplification using the following primers (forward primer: 5’-ATGATACGGCGACCACCGAGATCTACAC[5555555555]ACACTCTTTCCCTACACGACGCTCTTCCGATCT-3’, Reverse Primer: 5’-CAAGCAGAAGACGGCATACGAGAT[7777777777]GTGACTGGAGTTCAGACGTGTGCTCTTCCGATCT −3’, where sequences in parentheses are 10 bp custom Unique Dual Indexes). PCR reactions were cleaned using AxyPrep Mag PCR clean-up beads (Axygen Biosciences). This was followed by assessment of quality of the finished libraries using an Agilent 4200 TapeStation DNA 1000 kit (Agilent Technologies, Santa Clara, CA). Amount of DNAin the cleaned libraries were assessed using Qubit® dsDNA HS Assay Kit (ThermoFisher Scientific). Equimolar amounts of libraries were pooled prior to sequencing. For the construction and sequencing of the ITS libraries, samples were prepared in a similar manner to the one described in Illumina’s 16 s Metagenomic Sequencing Library Preparation Protocol, Part # 15,044,223 Rev. B (Illumina Inc., San Diego, California, USA) with the following modifications: The ITS region was amplified with fusion primers (forward primer: 5’- ACACTCTTTCCCTACACGACGCTCTTCCGATCTCTTGGTCATTTAGAGGAAGTAA−3’, reverse primer: 5’- GTGACTGGAGTTCAGACGTGTGCTCTTCCGATCTTCCTCCGCTTATTGATATGC−3’). Region specific primers were previously described (ITS1-F [38]; ITS4 [111]) and were modified to add Illumina adapter overhang nucleotide sequences to the region-specific sequences. Following initial amplification, reactions were cleaned using a 0.7 × volume of AxyPrep Mag PCR clean-up beads (Axygen Biosciences, Union City, CA). Using the initial amplification products as template, a second PCR was performed with primers that contain Illumina dual indexes and Sequencing adapters (Forward primer: 5’-AATGATACGGCGACCACCGAGATCTACAC[55555555]ACACTCTTTCCCTACACGACGCTCTTCCGATCT-3’, Reverse Primer: 5’-CAAGCAGAAGACGGCATACGAGAT[77777777]GTGACTGGAGTTCAGACGTGTGCTCTTCCGATCT −3’, where bracketed sequences are equivalent to the Illumina Dual Index adapters D501-D508 and D701-D712, N716, N718-N724, N726-N729). Following PCR, reactions were cleaned using a 0.7 × volume of AxyPrep Mag PCR clean-up beads (Axygen Biosciences). Quality and quantity of the finished libraries were assessed using an Agilent DNA 1000 kit (Agilent Technologies, Santa Clara, CA) and Qubit® dsDNA HS Assay Kit (ThermoFisher Scientific), respectively. Libraries were pooled in an equimolar fashion and appropriately diluted prior to sequencing. For both 16S and ITS, 300 bp paired end sequencing was performed using the Illumina NextSeq2000 as described here [38, 57, 111].

### Bioinformatic analysis of bacterial 16S sequence data

For 16S sequences, reads were denoised, joined, delineated into amplicon sequence variants (bacterial ASVs), and assigned taxonomy in the Qiime2 (v.2023.7) environment [20]. Forward and reverse primers were removed from paired end reads using cutadapt v 1.18 [72], demultiplexed, quality trimmed with a minimum Phred quality score of 20 [19] and denoised with *–p-trim-length 260* via q2-deblur in the deblur pipeline [6] to generate ASV. Bacterial ASVs with less than 30 reads or present in less than 3 samples within the dataset were removed to minimize potential errors in sequencing. The representative sequences were subsequently taxonomically classified using a classifier trained with the 99% OTU threshold using SILVA 138.1 database [90]. Afterwards, qiime phylogeny align-to-tree-mafft-fasttree generated the rooted phylogenetic tree of all the bacterial ASVs while sequences annotated as mitochondria and chloroplasts were excluded from subsequent analysis.

### Bioinformatic analysis of fungal ITS sequence data

Following the removal of ITS primers from sequences using cutadapt v 1.18 [72], denoising, chimera removal and fungal amplicon sequence variant (fungal ASV) determination for each data set was conducted using the DADA2 plugin in Qiime2 (v.2023.7) environment [20, 23] using a workflow specific for ITS sequences as described previously [22]. Taxonomic databases for classification were trained using a Naïve Bayes classifier for ITS with the q2-feature-classifier commands in Qiime2 using the developer’s QIIME-formatted UNITE v9.0 v25.07.2023 full-length dynamic dataset [1, 96]. Fungal ASVs assigned as *Viridiplantae* and *Protista* were removed and only reads assigned to Fungi were retained for use in downstream statistical analysis. Although some have argued against the use of ASVs for ITS sequences [55], we have opted to use fungal ASVs to maintain consistency with an earlier work [106].

### Statistical analyses

Downstream manipulation and analyses of the processed microbiome data generated from the bioinformatic analysis were carried out using phyloseq v.1.22.3 [74] and Qiime2R [17]. For alpha diversity, the vegan package [85] in R was used to perform and compute the diversity indices for both bacteria and fungi microbial communities. Kruskal–Wallis tests were performed to test for differences in alpha community diversity using Qiime2 and phyloseq in R. For beta diversity, the dissimilarity in species community composition between pairwise comparisons of bacterial and fungi communities were calculated and represented in Principal Coordinate Analysis (PCoA) ordination plots for Bray–Curtis, Unweighted Unifrac and Jaccard distances using the phyloseq package [74]. To see if beta diversity is statistically significant between the groups, Permutational multivariate analysis of variance (PERMANOVA, 999 permutations) tests which fits linear models to distance matrices and uses permutation test was used to compute variance explained by covariates genotype, location, and genotype by location interaction effects. Core microbiome was identified in R’s microbiome package [59] using a prevalence threshold of 50% with a detection limit of 0.001, while individual ASV plots (bacterial and fungal ASVs) for each core microbiome was estimated using centered log ratio as described earlier [17]. In addition, taxa bar plots of the top 30 most abundant phyla at appropriate taxonomic levels was generated to give an indication of how dominant phyla change between genotypes and across locations. Linear discriminant analysis (LDA) effect size which estimates magnitude of differential abundance for pairwise genotype and location comparison was conducted using lefser package in R [99] and visualized in dot plots. In addition, microbial taxa that are differentially abundant between genotypes and locations were performed using edgeR package in R [97], which uses a TMM normalization method and negative binomial model to model raw counts of sequences observed for bacterial and fungal ASVs. Using exact test in edgeR, the log2-fold change in abundance for each bacterial and fungal ASVs across all pairwise contrasts was performed following adjustment for covariates. Significance was determined at *p* < 0.05 after adjustment for multiple comparisons using the Benjamini–Hochberg false discovery rate. Venn diagrams were conducted by R package VennDiagram (v1.7.1, https://CRAN.R-project.org/package=VennDiagram). UpSet plots were generated by R package UpSetR [27], while transformed centered log ratio (CLR) used to address the compositional nature of the microbiome data was used for bacterial and fungal ASV abundances plots and were performed using Qiime2R [17]. We carried out two association analyses in this study using lme4 package in R [11]. For the association analysis between malt quality traits and alpha diversity metrics (bacterial and fungal), linear mixed effect model was used to control for variation due to random effects (genotype and location) while using bacterial and fungal alpha diversity indices (Shannon and Faith’s phylogenetic diversity for bacteria and Shannon and Simpson for fungi) as fixed effects. For the association analysis between each fungal and bacterial genus taxa (total abundances) and malt quality traits, linear mixed effect model was used to assess the nature of the relationship between each genus (bacterial and fungal abundance) and malt quality traits following adjustments for covariates. Multiple hypothesis correction for *p* values was performed using the Benjamini–Hochberg method of False Discovery Rate (FDR) control for the association analysis [12]. Additionally, correlation matrix with significance levels between alpha diversity metrics (bacterial and fungal) and malt quality traits was performed and visualized using R version 4.3.0 [91]. Also, genotype-location effects on malt quality and alpha diversity indices was performed using the one-way ANOVA, followed by Tukey’s post hoc test in R version 4.3.0 [91].

## Results

### Sequencing summary

For 16S sequencing, a total of 32,016,711 raw sequences were obtained. The lowest and the highest number of reads per sample were 274,889 and 747, 397 respectively with reads from other samples nestled in-between. After adaptor, primer, and chimera removal including quality and length trimming, 8,551,980 high-quality reads clustered at 99% sequence identity resulted in 1,554 bacterial ASVs. Bacterial ASVs annotated as mitochondria and chloroplast were removed, which resulted in 877 unique ASVs from 6,888,642 sequence features and were subsequently used for downstream analysis. To compare the diversity in different samples, we rarefied the data to 55,907 reads per sample and based on rarefaction curves, the sequencing depth was sufficient to capture the bacterial seed endophytic diversity (Supplementary Figure S1A). Similarly for the fungal ITS sequencing, a total of 31,110,549 raw sequences were observed. After adaptor, primer, and chimera removal including quality and length trimming, 25, 971,339 high-quality reads clustered at 99% sequence identity resulted in 18,110 fungal ASVs. To compare the diversity in different samples, we rarefied the data and based on rarefaction curves, the sequencing depth was sufficient to capture the fungal microbial diversity (Supplementary Figure S1B).

### Bacterial and fungal communities in relation to barley cultivar and location

**Fig. 1:**
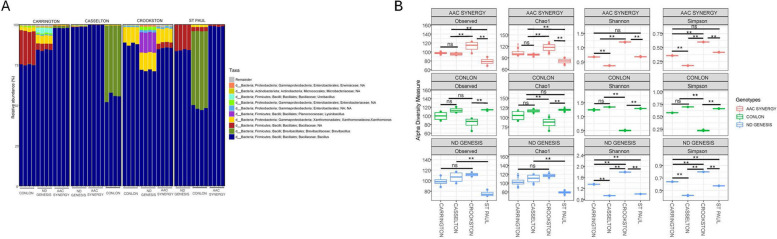
Taxonomic summary and bacterial diversity analyses of seed endophytes of three malting genotype/cultivars grown across four locations. **A** Stacked bar chart of the taxonomic composition of bacterial communities of different genotype-location sample types aggregated at the genus level. Each stacked column represents an independent sample (*n* = 48). Only the top 10 most abundant taxa were colored individually, the rest are colored in gray and listed as “Remainder”. Samples are clustered by cultivar types (Conlon, ND Genesis and AAC Synergy) and location (Carrington, Casselton, Crookston and St Paul); **B** The bacterial seed endophytes alpha-diversity indices based on observed species richness, Chao1, Shannon diversity index, and Simpson varied for each malting cultivar/genotype across locations (ANOVA for all the alpha diversity indices were *p* ≤ 0.01). Significant asterisks ‘**’ (*p*** ≤ **0.01), ns = non-significant. Actual values of alpha-diversity indices can be seen in Table 1

**Fig. 2:**
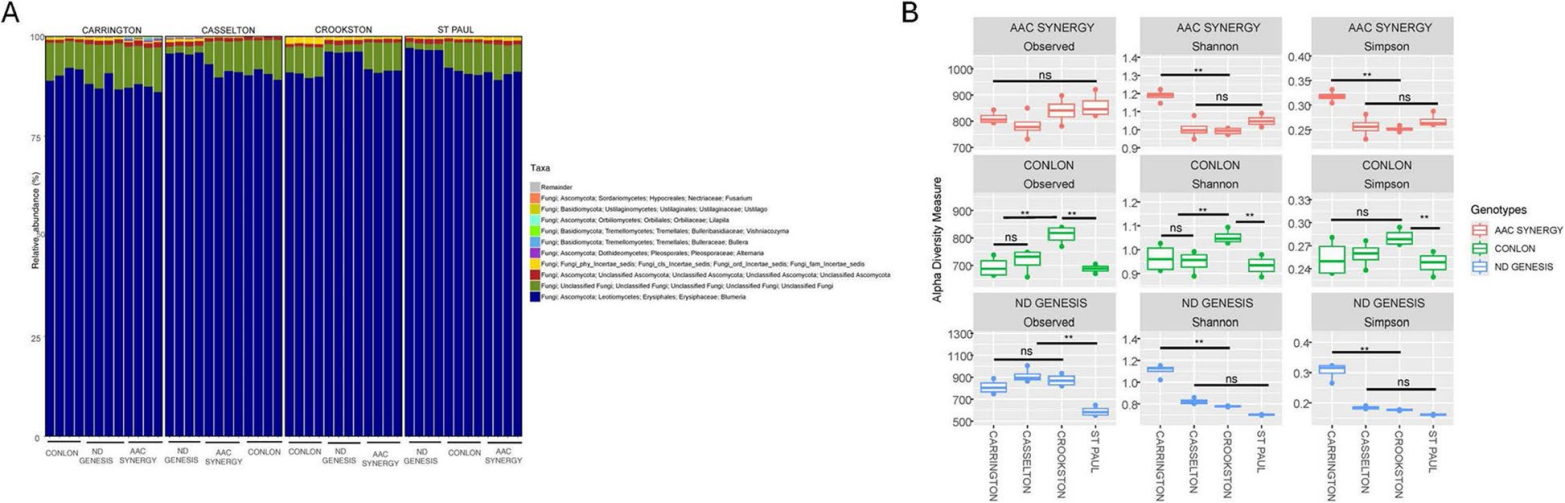
Taxonomic summary and fungal diversity analyses of seed endophytes of three malting barley genotype/cultivars grown across four locations. **A** Stacked bar chart of the taxonomic composition of fungi communities of different genotype-location sample types aggregated at the genus level. Each stacked column represents an independent sample (*n* = 48). Only the top 10 most abundant taxa were colored individually, the rest are colored in gray and listed as “Remainder”. Samples are clustered by cultivar types (Conlon, ND Genesis and AAC Synergy) and location (Carrington, Casselton, Crookston and St Paul); **B** The fungal seed endophytes alpha-diversity indices based on observed species richness, Shannon diversity index, and Simpson varied for each malting cultivar/genotype across locations (ANOVA for all the alpha diversity indices were significant). Significant asterisks ‘**’ (*p*** ≤ **0.01), ns = non-significant. Actual values of alpha-diversity indices can be seen in Table 2

The predominant bacterial taxonomic composition of seed endophytes of malting barley genotypes (Conlon, ND Genesis, and AAC Synergy) across locations (Carrington, Casselton, Crookston, and St. Paul) can be seen in Fig. 1. Three major phyla, Proteobacteria, Actinobacteria, and Firmicutes were identified among the bacterial seed endophytes, with firmicutes observed to be the most abundant (Supplementary Table S1). Taxonomic classification at the genus level identified genus *Bacillus* as the most abundant and ranked among the top 10 genera (Fig. 1A). For within-location comparison across genotypes, we observed certain microbial taxa are more prevalent in certain genotypes than others. For example, *Xanthomonas* and *Ureibacillus* genera were more abundant in ND Genesis, compared to Conlon and AAC Synergy seed samples from Carrington location (Fig. 1A, Supplementary Table S2). Similarly, for Casselton and St Paul locations, genus *Brevibacillus* was more abundant in Conlon genotype compared to AAC Synergy and ND Genesis. In addition, genus *Lysinibacillus* was most abundant in ND Genesis seed samples from Crookston compared to the rest of the genotype-location, while genus *Xanthomonas* was surprisingly more abundant for all the genotypes in Crookston compared to other genotype-location (Fig. 1A, Supplementary Table S2). For the taxonomic composition of fungal communities, nine phyla were identified with phylum Ascomycota found to be the most abundant (Supplementary Table S3). Taxonomic classification at the genus level identified *Blumeria* as the most abundant (Supplementary Table S4) among the top 10 genera (Fig. 2A).

Alpha diversity measures (Observed species, Chao1, Shannon and Simpson) for assessing the microbial diversity of seed endophytes based on genotype-location comparison indicated that for bacterial diversity for Observed species (number of ASVs/species when samples are rarefied) and Chao1 (measure of species richness), highest values (114 ± 1.22 for Observed species and 119.35 ± 2.66 for Chao1 index) were found in Conlon-St. Paul and was significantly different from the lowest richness detected in ND Genesis-St. Paul samples (75.5 ± 4.72 for Observed species and 78.87 ± 3.56 for Chao1 index); values for the remaining genotype-location samples are nestled between the two extremes (Table 1). Shannon and Simpson indices (a metric used to estimate species diversity) and Faith’s phylogenetic diversity (Faith’s PD) which consider phylogeny to estimate diversity indicated that ND Genesis-Casselton samples exhibited significantly higher values for Shannon and Faith’s PD (2.98 ± 0.55 for Shannon; 2.17 ± 0.12 for Faith’s PD) compared to the remaining samples. In addition, significantly lower alpha diversity values were detected in AAC Synergy-Casselton samples for both Shannon and Simpson indices (0.37 ± 0.008 for Shannon; 0.18 ± 0.0002 for Simpson) and ND Genesis-St. Paul for Faith’s PD (0.93 ± 0.24) compared to the remaining samples (Table 1). It was apparent that AAC Synergy and ND Genesis genotypes consistently showed similar patterns for all the alpha diversity measures and behaved differently from Conlon (Fig. 1B). For fungal communities, alpha diversity measures (Observed species, Shannon, and Simpson) showed that for Observed species, ND Genesis-Casselton had the highest value (914 ± 54.97), which was significantly different from the lowest value detected in ND Genesis-St. Paul (590.5 ± 38.24). Similarly, AAC Synergy-Carrington samples had the highest values for Shannon and Simpson index (1.19 ± 0.027 for Shannon; 0.32 ± 0.01 for Simpson) and was significantly different from the lowest values detected in ND Genesis-St. Paul (0.7 ± 0.003 for Shannon; 0.16 ± 0.001 for Simpson). Notably, lower alpha diversity values were consistently detected in ND Genesis-St. Paul for Observed species, Shannon, and Simpson indices (Table 2). Fungal diversity across locations for AAC Synergy and ND Genesis showed similar pattern for Shannon and Simpson index except for Observed species which behaved differently (Fig. 2B).

**Table 1:** Alpha diversity statistics for barley seed endophytic bacterial communities (mean ± S.D)

Genotype-location	Observed	Chao1	Shannon	Simpson	Faith_pd
ND GENESIS-CASSELTON	106.5 ± 9.23^a^	109.8 ± 8.84^a^	2.98 ± 0.55^a^	0.42 ± 0.005^d^	2.17 ± 0.12^a^
AAC SYNERGY-CASSELTON	95.5 ± 2.5^b^	97.88 ± 2.84^ab^	0.37 ± 0.008^f^	0.18 ± 0.002^ g^	1.49 ± 0.08^e^
CONLON-CASSELTON	113.3 ± 5.21^a^	117.16 ± 3.54^a^	1.35 ± 0.005^c^	0.7 ± 0.001^b^	1.7 ± 0.19^c^
CONLON-CROOKSTON	83.5 ± 11.46^b^	85.6 ± 13.31^b^	0.5 ± 0.018^f^	0.22 ± 0.01^f^	1.84 ± 0.09^bc^
ND GENESIS-CROOKSTON	111.8 ± 3.03^a^	117.2 ± 3.45^a^	1.79 ± 0.009^b^	0.79 ± 0.002^a^	1.83 ± 0.02^bc^
AAC SYNERGY-CROOKSTON	112.5 ± 10.4^a^	116.46 ± 10.04^a^	1.20 ± 0.011^d^	0.6 ± 0.004^c^	1.92 ± 0.08^b^
ND GENESIS-ST PAUL	75.5 ± 4.72^c^	78.87 ± 3.56^c^	1.01 ± 0.003^d^	0.58 ± 0.003^b^	0.93 ± 0.24^ g^
CONLON-ST PAUL	114 ± 1.22^a^	119.35 ± 2.66^a^	1.29 ± 0.009^c^	0.66 ± 0.004^b^	1.54 ± 0.16^d^
AAC SYNERGY-ST PAUL	78.75 ± 7.29^c^	81.5 ± 6.89^b^	0.69 ± 0.012^e^	0.42 ± 0.004^d^	1.19 ± 0.07^f^
CONLON-CARRINGTON	99.75 ± 8.93^a^	104.52 ± 11.07^a^	1.24 ± 0.022^c^	0.58 ± 0.004^b^	1.8 ± 0.07^bc^
ND GENESIS-CARRINGTON	98.5 ± 7.83^a^	102.82 ± 9.46^a^	1.35 ± 0.010^c^	0.64 ± 0.002^b^	1.95 ± 0.08^b^
AAC SYNERGY-CARRINGTON	97.5 ± 2.5^b^	103.39 ± 7.6^ab^	0.68 ± 0.0073^e^	0.36 ± 0.004^d^	1.64 ± 0.07^ cd^

Means in the same column bearing different superscript letters differ significantly (*P* < 0.05)

**Table 2:** Alpha diversity statistics for barley seed endophytic fungal communities (mean ± S.D)

Genotype-location	Observed	Shannon	Simpson
ND GENESIS-CASSELTON	914 ± 54.97^a^	0.82 ± 0.022^c^	0.18 ± 0.004^c^
AAC SYNERGY-CASSELTON	784.25 ± 42.54^a^	1.01 ± 0.05^b^	0.26 ± 0.017^bc^
CONLON-CASSELTON	717.5 ± 36.75^b^	0.95 ± 0.04^b^	0.26 ± 0.013^b^
CONLON-CROOKSTON	811 ± 28.7^a^	1.05 ± 0.026^ab^	0.28 ± 0.009^b^
ND GENESIS-CROOKSTON	873.25 ± 47.52^a^	0.78 ± 0.004^c^	0.18 ± 0.001^c^
AAC SYNERGY-CROOKSTON	840.25 ± 42.38^a^	0.99 ± 0.016^b^	0.25 ± 0.005^bc^
ND GENESIS-ST PAUL	590.5 ± 38.24^c^	0.7 ± 0.003^c^	0.16 ± 0.001^c^
CONLON-ST PAUL	688 ± 12.98^b^	0.93 ± 0.036^b^	0.25 ± 0.013^bc^
AAC SYNERGY-ST PAUL	858.25 ± 39.57^a^	1.05 ± 0.028^ab^	0.27 ± 0.011^bc^
CONLON-CARRINGTON	694.5 ± 31.12^b^	0.96 ± 0.05^b^	0.25 ± 0.02^b^
ND GENESIS-CARRINGTON	811.25 ± 54.66^a^	1.1 ± 0.05^a^	0.30 ± 0.02^a^
AAC SYNERGY-CARRINGTON	812 ± 19.72^a^	1.19 ± 0.027^a^	0.32 ± 0.01^a^

Means in the same column with different superscript letters differ significantly (*P* < 0.05)

**Fig. 3:**
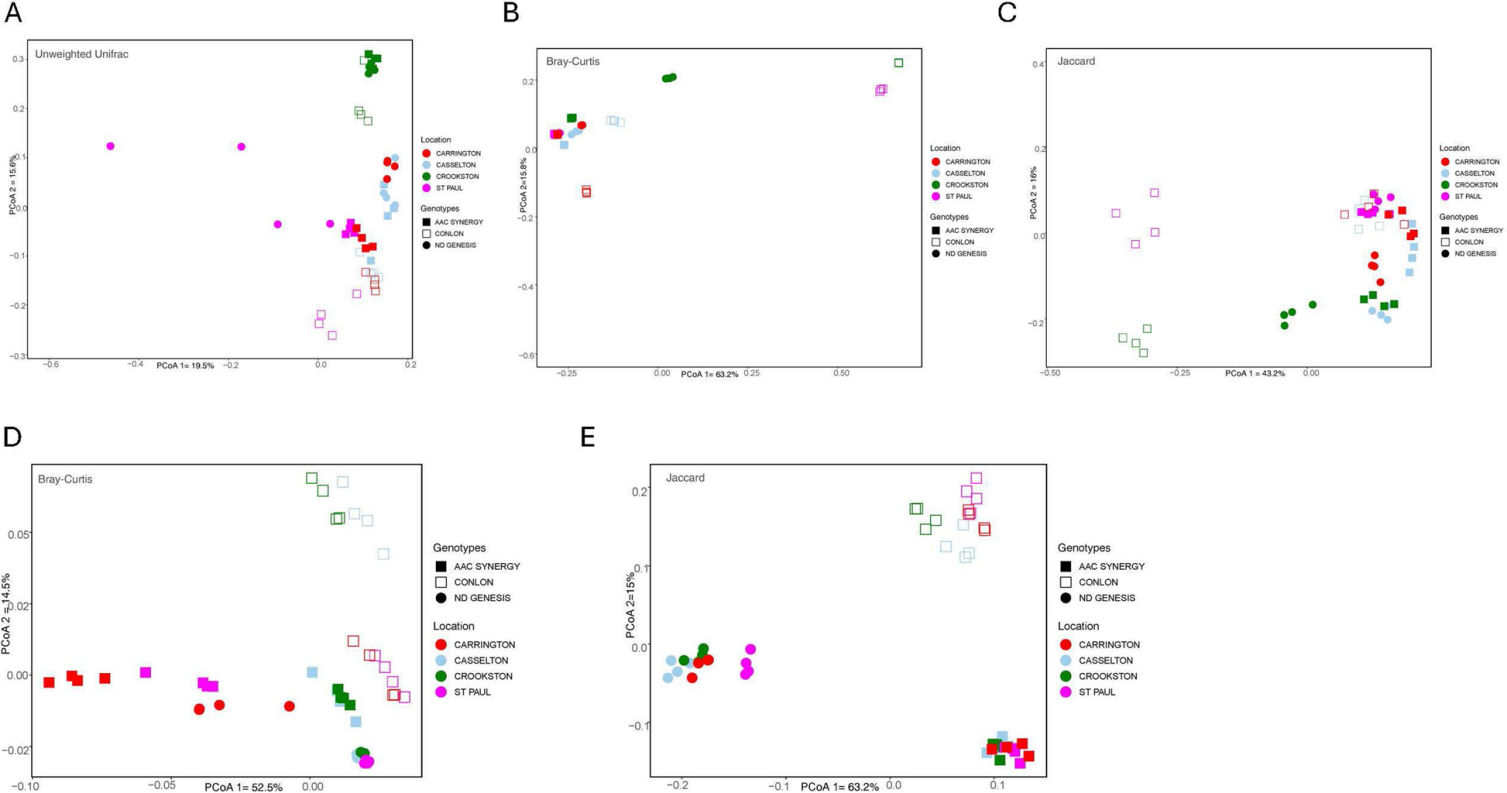
Bacterial and fungal communities of malting barley seed endophytes in relation to cultivar and location. **A-C** represents the PCoA of 16S rRNA amplicon sequencing data while **D** and** E** represents PCoA of ITS amplicon sequencing data of genotype-location samples based on unweighted unifrac (**A**), Bray–Curtis (**B**,** D**) and Jaccard distances (**C**, **E**). Each shape and color represent one replicate sample for genotype and location respectively for bacterial and fungal communities. Adonis in vegan package in R verified significant differences between the genotypes and locations (*p* ≤ 0.001). Pairwise PERMANOVA estimates are presented in Table 3 (16 s) and 4 (ITS)

In order to investigate and visualize the seed endophytic microbiome for sample dissimilarity, we performed beta diversity measures, including Bray–Curtis distance for quantitative compositional changes (Bray–Curtis considers species abundance and species presence or absence), Jaccard distance for qualitative composition changes (Jaccard distance is based on species presence or absence) and Unweighted UniFrac distance for phylogenetic changes (Unweighted Unifrac considers presence/ absence and phylogenetic relationships). For the beta diversity of the seed bacterial endophytes microbiome, all barley genotypes from Crookston location clustered separately from the other genotype-location samples for Unweighted Unifrac, and to a certain degree for Bray–Curtis and Jaccard distances based on the two-dimension PCoA plots (Fig. 3A-C; Supplementary Figure S2). For the fungal communities, distinct clustering along barley genotypes irrespective of location was observed for Jaccard distance and to a similar extent for Bray–Curtis distance (Fig. 3D and E); an exception to this was for ND Genesis where samples from Carrington clustered separately from other locations for Bray–Curtis (Supplementary Figure S3A). Permutational multivariate analysis of variance (PERMANOVA) for bacterial communities revealed significant variability among barley genotypes, with 16% in phylogeny, 35% in quantitative composition, and 27% in qualitative composition. Similarly, significant variability among barley genotypes across locations were observed with 30% in phylogeny, 26% in quantitative composition, and 27% in qualitative composition (Table 3). For fungal communities, barley genotypes showed significant variability, with 29% in quantitative composition and 27% in qualitative composition and for location, we observed a significant variability of 28% in quantitative composition and 27% in qualitative composition (Table 4). Notably, genotype and location interactions were significant for Bray–Curtis (PERMANOVA *R*^2^ = 0.35 for bacterial, *R*^2^ = 0.29 for fungal; *p* ≤ 0.001), Jaccard (PERMANOVA *R*^2^ = 0.29 for bacterial, *R*^2^ = 0.29 for fungal; *p* ≤ 0.001) and Unweighted Unifrac distances (PERMANOVA *R*^2^ = 0.28 for bacterial; *p* ≤ 0.001) when all samples were jointly considered for bacterial (Supplementary Table S5) and fungal communities (Supplementary Table S6). In summary, these results indicated that barley genotypes, location, and their interactions significantly influenced both bacterial and fungal alpha and beta diversities and influenced the assembly of the seed endophytic microbiome.

**Table 3:** PERMANOVA of bacterial seed endophytes based on Unweighted Unifrac, Bray–Curtis and Jaccard distances

	Unweighted Unifrac distances for 16S		Bray–Curtis distances for 16S		Jaccard distances for 16S	
PERMANOVA	R^2^	*p* ≤	R^2^	*p* ≤	R^2^	*p* ≤
Genotype	0.16	0.001	0.35	0.001	0.27	0.001
ND Genesis vs AAC Synergy	0.10	0.002	0.20	0.001	0.17	0.001
ND Genesis vs Conlon	0.11	0.001	0.26	0.001	0.21	0.001
AAC Synergy vs Conlon	0.16	0.001	0.35	0.001	0.30	0.001
Location	0.30	0.001	0.26	0.001	0.27	0.001
Casselton vs Crookston	0.28	0.001	0.35	0.001	0.28	0.001
Casselton vs Saint Paul	0.18	0.001	0.09	0.116	0.13	0.053
Casselton vs Carrington	0.22	0.001	0.13	0.025	0.13	0.019
Crookston vs Saint Paul	0.21	0.001	0.11	0.047	0.13	0.046
Crookston vs Carrington	0.26	0.001	0.30	0.001	0.22	0.002
Saint Paul vs Carrington	0.19	0.001	0.13	0.097	0.13	0.043

**Table 4:** PERMANOVA of fungal seed endophytes based on Bray–Curtis and Jaccard distances for ITS

	Bray–Curtis distances for ITS		Jaccard distances for ITS	
PERMANOVA	R^2^	*p* ≤	R^2^	*p* ≤
GENOTYPE	0.29	0.001	0.27	0.001
ND GENESIS versus AAC SYNERGY	0.24	0.001	0.23	0.001
ND GENESIS versus CONLON	0.18	0.001	0.17	0.001
AAC SYNERGY versus CONLON	0.26	0.001	0.25	0.001
LOCATION	0.28	0.001	0.27	0.001
CASSELTON versus CROOKSTON	0.05	0.02	0.06	0.009
CASSELTON versus ST PAUL	0.38	0.001	0.35	0.001
CASSELTON versus CARRINGTON	0.17	0.001	0.16	0.001
CROOKSTON versus ST PAUL	0.40	0.001	0.37	0.001
CROOKSTON versus CARRINGTON	0.15	0.001	0.15	0.001
ST PAUL versus CARRINGTON	0.08	0.002	0.09	0.001

### Analysis of alpha diversity metrics and microbial (bacterial and fungal) genera in relation to malt quality traits

**Fig. 4:**
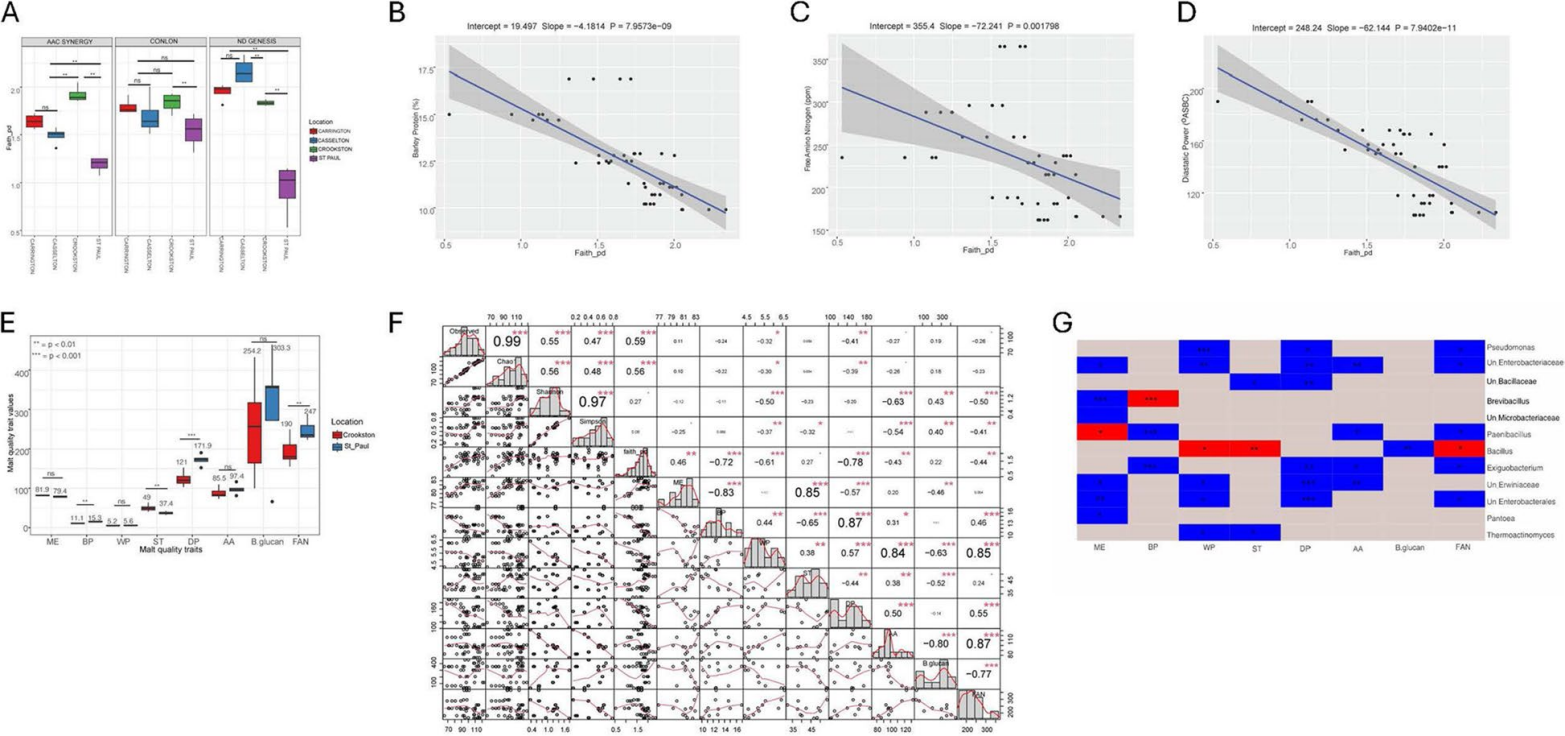
Association analysis between bacterial alpha diversity, along with bacterial genus taxa, and malt quality traits. **A** Alpha diversity metric (Faith_pd) of bacterial seed endophytes of three malting barley genotype/cultivars grown across four locations. (**B-D**) Negative relationship between Faith_pd and barley protein (BP), **B**, free amino nitrogen (FAN) **C**, diastatic power (DP) **D** based on linear regression after adjusting for covariates genotype and location; **E** Malt quality analyses of Conlon, ND Genesis and AAC Synergy from Crookston and St. Paul that were statistically assessed using Welch test (*N* = 24, 15 samples from Crookston and 9 samples from St. Paul). **F** Correlation analysis between alpha diversity metrics and malt quality traits. (**G**) Association analyses between bacterial taxa (genus level) and malt quality traits after adjusting for genotype and location effects in a linear mixed model. Only genera significantly associated with malt quality traits were shown. Red color indicates significant positive association; blue color indicates significant negative association; grey color indicates no association. Significant asterisks ‘*’ indicates *p*** ≤ **0.05; ‘**’ indicates *p*** ≤ **0.01; ‘***’ indicates *p*** ≤ **0.001. Un = Unclassified

**Fig. 5:**
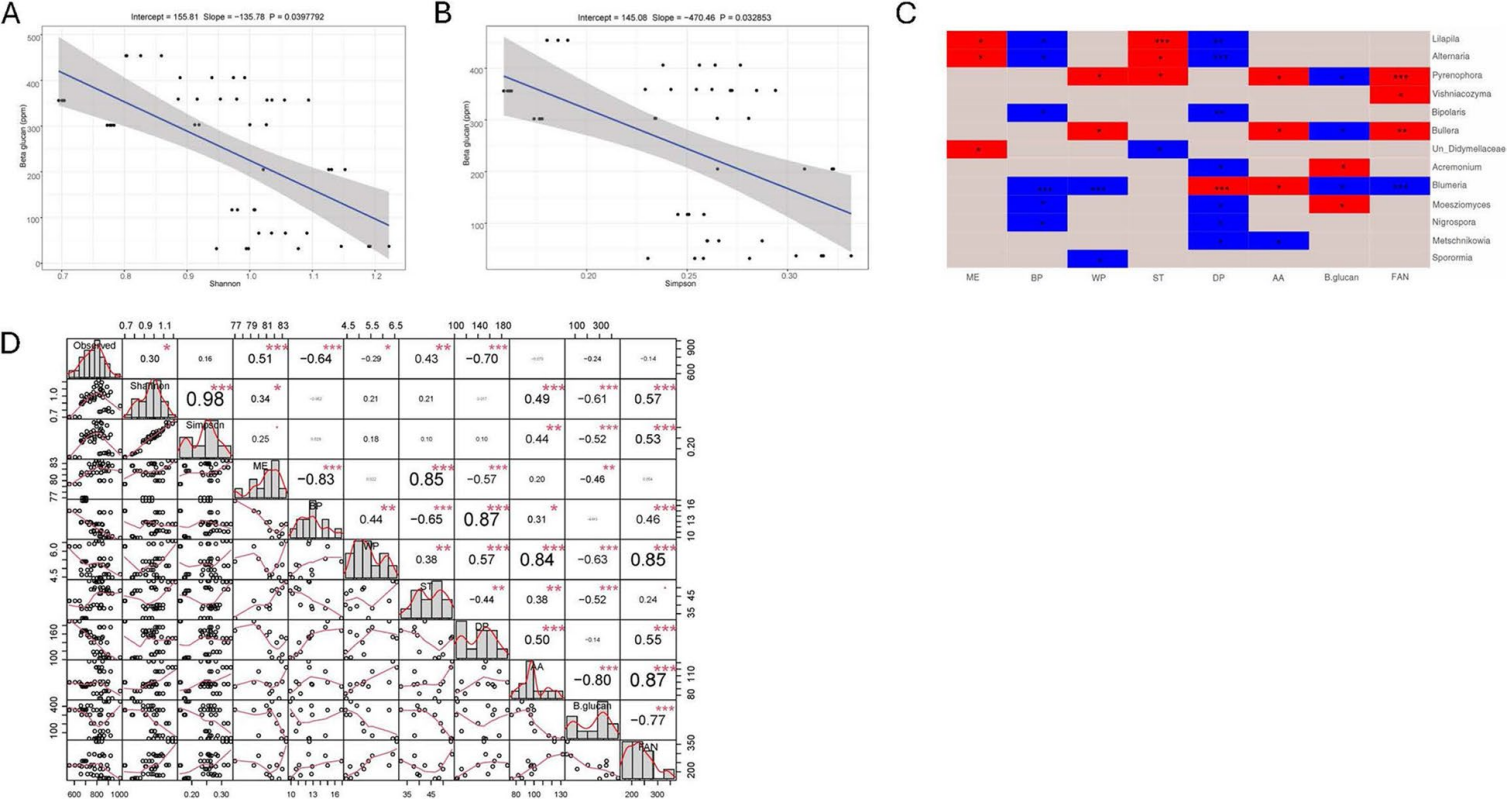
Association analysis between fungal alpha diversity, along with fungal genus taxa and malt quality traits. **A** Shannon index and **B** Simpson index **B** and their negative association with beta glucan after adjusting for covariates genotype and location effects. **C** Relationship between fungal taxa (genus level) and malt quality traits after adjusting for genotype and location effects in a linear mixed model. Only genus significantly associated with malt quality traits were shown. Red color indicates significant positive association; blue color indicates significant negative association; grey color indicates no association. **D** Correlation analysis between alpha diversity metrics and malt quality traits. Significant asterisks ‘*’ indicates *p*** ≤ **0.05; ‘**’ indicates *p*** ≤ **0.01; ‘***’ indicates *p*** ≤ **0.001. Un = Unclassified

The genotype-location effects on malt quality traits are detailed in Supplementary Table S10. To test our hypothesis that bacterial and fungal genera, along with alpha diversity indices, are associated with malt quality traits, we examined alpha diversity indices (Faith’s PD and Shannon for bacteria; Shannon and Simpson for fungi) and their associations with malt quality traits, including their respective genera (Figs. 4 and 5). For bacterial communities, we observed that Faith’s PD was significantly negatively correlated with malt quality traits- BP (Fig. 4B), FAN (Fig. 4C), DP (Fig. 4D), while Shannon index was significantly negatively correlated with FAN (Supplementary Figure S4A) and AA (Supplementary Figure S4B). Further analysis revealed a similar pattern of association with BP (*r* = −0.72; *p* ≤ 0.001) and DP (*r* = −0.78; *p* ≤ 0.001) showing the strongest correlations with Faith’s PD, and AA (*r* = −0.63; *p* ≤ 0.001) showing moderate to high correlations with Shannon index (Fig. 4F). Given the negative correlation between alpha diversity indices and malt quality, we asked whether this pattern of relationship would be detected when applied on a separate malt quality data of barley samples grown in Crookston and St. Paul, MN. We used malt quality data for crop year 2022 for St. Paul and Crookston because of the significant variability between St. Paul and Crookston for all the three genotypes (Fig. 4A). Based on Welch’s t-test (due to unequal sample sizes), we found that the malt quality data for these two locations mirrors our previous findings that showed that higher alpha diversity indices (Faith’s PD and Shannon index) are negatively correlated with some malt quality traits. Interestingly, and as speculated, Crookston with significantly higher Faith’s PD had lower malt BP, DP, and FAN (Fig. 4E). While AA showed significant negative relationship with Shannon index (Fig. 4F), the malt quality data showed no significant difference between Crookston and St. Paul samples although Crookston AA were lower than St. Paul (Fig. 4E). Notably, significantly low to moderate correlations existed between alpha diversity indices and other malt quality traits (Fig. 4F). For the association between genera and malt quality traits, out of 37 genera that were identified in this study (Supplementary Table S2), *Brevibacillus* (associated with BP), *Paenibacillus* (associated with ME), and *Bacillus* (associated with ST, WP, and FAN) had significant positive associations, while the remaining significant genera were negatively correlated (Fig. 4G).

For the fungal communities, only β-D-glucan showed a significant negative correlation with the Shannon and Simpson diversity indices (Fig. 5A and B). This observation is consistent with the correlation analysis, which showed a significant negative correlation between β-D-glucan and Shannon index (*r* = −0.61; *p* ≤ 0.001) as well as the Simpson index (*r* = −0.52; *p* ≤ 0.001) (Fig. 5D). Although, FAN and AA are significantly correlated with Shannon and Simpson indices (Fig. 5D), those correlations were not significant after adjusting for covariates genotype and location. For the association between fungal genera and malt quality traits, out of 113 genera (Supplementary Table S4), thirteen fungal genera were significantly associated with malt quality traits. Malt quality traits BP, WP, β-D-glucan and FAN were significantly negatively associated with genus *Blumeria*, while AA and DP were significantly positively associated (Fig. 5C). In summary, both bacterial and fungal alpha diversities, along with certain bacterial and fungal genera, are significantly associated with malt quality.

### Analyses of the core microbiome at the genus level and microbial (bacterial and fungal) ASVs in relation to barley cultivar and location

**Fig. 6:**
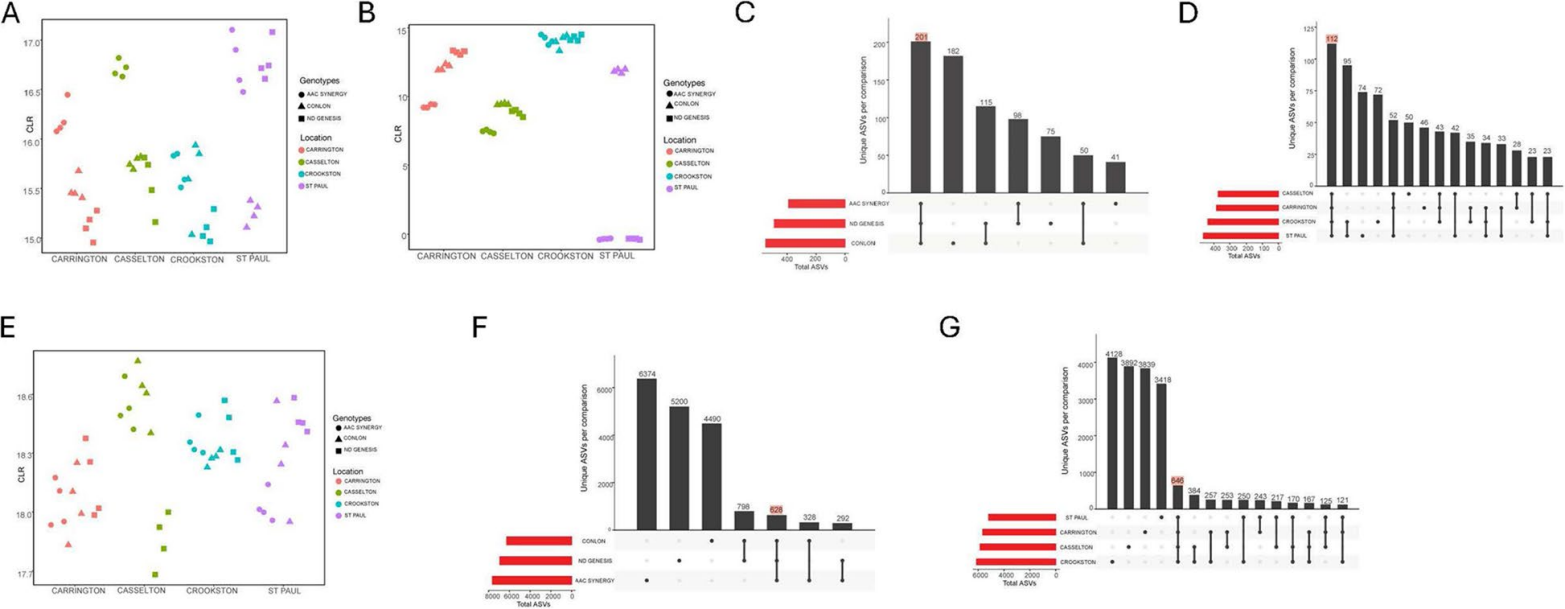
Abundance and UpSet plots of bacterial and fungal seed endophytes in relation to cultivar and location. Centered log ratio of ASV abundances for genus Bacillus **A** and Xanthomonas **B** among cultivars across location. **C** UpSet plot showing the number of bacterial ASVs that are shared between or are unique for AAC Synergy, ND Genesis and Conlon. **D** UpSet plot showing the number of bacterial ASVs that are shared between or are unique for Casselton, Carrington, Crookston and St Paul. **E** Centered log ratio of ASV abundances for genus Blumeria among cultivars across location. **F** UpSet plot showing the number of fungal ASVs that are shared between or are unique for AAC Synergy, ND Genesis and Conlon. **G** UpSet plot showing the number of fungal ASVs that are shared between or are unique for Casselton, Carrington, Crookston and St Paul. For **C, D, F, G**, number in color indicates number of shared ASVs common across all cultivar or location

We defined a core microbiome as a set of microbes that are prevalent and consistently detected within a given threshold (prevalence threshold of 50% with a detection limit of 0.001) in our samples. For the bacterial core, genera *Bacillus* (detected in all 48 samples) and *Xanthomonas* (detected in 40 samples) were above the set threshold (Fig. 6A and B) and were among the top 30 bacterial seed endophytes (Supplementary Figure S6). For the fungal core, only genus *Blumeria* (detected in all 48 samples) was above the set threshold (Fig. 6E) and the most abundant among the top 30 microbial taxa (Supplementary Figure S7). Further, BlastN analysis of bacterial genus *Xanthomonas* revealed that the 16 s rRNA partial sequence (~ 260 bp) shared 100% homology with *Xanthomonas translucens* (Supplementary Figure S8), a seed-borne phytopathogen that causes a plant disease called bacterial leaf streak. Also notable was the prevalence of Xanthomonas in all locations except ND Genesis and AAC Synergy from St. Paul location (Fig. 6B).

We then focused on shared and unique bacterial and fungal ASVs between seed endophytic microbiome of all genotypes and locations. A total of 390, 489, and 548 unique bacterial ASVs were detected in AAC Synergy, ND Genesis, and Conlon seed samples, respectively. Whereas 77% ((201 + 98)/390) of the bacterial ASVs on AAC Synergy were shared with those on ND Genesis, only 65% ((201 + 115)/489) and 64% ((201 + 50)/390) of the bacterial ASVs on Conlon were shared with ND Genesis and AAC Synergy, respectively (Fig. 6C). Interestingly, we found 14% (201/1427) of the bacterial ASVs present in all the three barley genotypes while 6.8% (112/1658) of the bacterial ASVs were shared across all four locations (Fig. 6D). Also, we investigated core bacterial ASVs for each genotype for all locations and we observed that 1.2% (10/835), 4% (33/832) and 8.1% (63/778) of the bacterial ASVs for Conlon, ND Genesis and AAC Synergy respectively, were shared across all locations (Supplementary Figure S5A-C) indicating a higher proportion of bacterial ASVs for ND Genesis and AAC synergy is retained compared to Conlon irrespective of location. Analysis of the fungal communities showed that a total of 7622, 6918, and 6244 unique fungal ASVs were detected in AAC Synergy, ND Genesis, and Conlon seed samples, respectively. Whereas 12% (628 + 292)/7622) of the fungal ASVs on AAC Synergy were shared with those on ND Genesis, 21% (798 + 628)/6918) and 13% (628 + 328)/7622) of the fungal ASVs on Conlon were shared with ND Genesis and AAC Synergy, respectively (Fig. 6F). Only 3% (628/20,784) of the core fungal ASVs were present in all the three barley genotypes while 2.8% (646/22,818) of the fungal ASVs were shared across all four locations (Fig. 6G). Also, we investigated core fungal ASVs for each genotype for all locations and observed that 4% (323/8050), 4% (346/8991) and 3.4% (326/9593) of the fungal core ASVs for Conlon, ND Genesis, and AAC Synergy respectively, were shared across all locations (Supplementary Figure S5D-F). In summary, the proportion of shared and unique ASVs across genotypes and locations differ for both bacterial and fungal communities.

### Differential abundance analysis based on pairwise comparisons between genotypes and locations

Key factors driving the observed shifts in microbiome composition identified to date include intrinsic host genotypic traits and external environmental conditions [15]. Differential abundance analysis was used to investigate how genotype and location shape the overall microbial community structure (Supplementary Table S7 and S8). Pairwise comparison between St. Paul and Crookston location showed that 11 bacterial ASVs belonging to phyla Firmicutes, Actinobacteria, and Proteobacteria were significantly enriched or depleted, with Firmicutes having higher log FC for both enriched and depleted bacterial ASVs (Supplementary Figure S11A). Similarly, 29 bacterial ASVs were significantly enriched for Carrington but surprisingly, no bacterial ASVs were enriched in Casselton (Supplementary Table S7), with phyla Proteobacteria, Actinobacteria, Firmicutes and Bacteroidota significantly impacted in abundance in Carrington and Casselton location (Supplementary Figure S11B). Out of 11 differentially abundant bacterial ASVs for Crookston and St Paul and 29 for Casselton and Carrington, 9 were shared (Supplementary Figure S11C, Supplementary Table S9). For genotype pairwise comparison, we observed 8 (Conlon and AAC Synergy; Supplementary Figure S11D), 10 (ND Genesis and Conlon; Supplementary Figure S11E), and 3 (ND Genesis and AAC Synergy; Supplementary Figure S11F) bacterial ASVs that were significantly impacted in abundance, with more than half belonging to Firmicutes based on the three pairwise genotype comparisons (Supplementary Figure S11D-F; Supplemental Table S7). While no bacterial ASVs were shared among the three pairwise genotype comparison, only 3 bacterial ASVs were shared between ND Genesis and Conlon and ND Genesis and AAC Synergy (Supplementary Figure S11G). Interestingly, some of the bacterial ASVs identified in the location and genotype-based pairwise differential abundance analysis were also detected using linear discriminant analysis for investigating microbial community structure (Supplementary Figure S9). For fungal communities, 13 fungal ASVs were significantly enriched or depleted between Carrington and Casselton location and St. Paul and Crookston location (Supplementary Figure S12A and Supplementary Figure S12B) but only 3 fungal ASVs were shared between both location comparison (Supplementary Figure S12C, Supplementary Table S9). Similarly, 16 (Conlon and AAC Synergy; Supplementary Figure S12D), 21 (ND Genesis and Conlon; Supplementary Figure S12E), and 9 (ND Genesis and AAC Synergy; Supplementary Figure S12F) fungal ASVs were significantly enriched and depleted in abundance and were associated with phyla Ascomycota, Basidiomycota and Unclassified fungi based on the three pairwise genotype comparisons (Supplementary Figure S12D-F; Supplementary Table S8). While just one single fungal ASVs was shared among the three pairwise genotype comparison, we observed a higher number of shared fungal ASVs of pairwise genotype comparison with Conlon (11 shared fungal ASVs between Conlon and AAC Synergy and ND Genesis and Conlon) than without Conlon (Supplementary Figure S12G). Interestingly, some of the fungal ASVs identified in the differential abundance analysis were also detected using linear discriminant analysis (Supplementary Figure S10).

## Discussion

Seed endophytes can be transmitted from parents to progeny and contain diverse microbial communities whose role in boosting crop yields are of particular interest for researchers. Barley seed microbiome not only perform key functions in germination, growth, biotic and abiotic stress protection [75], but their reported role in influencing malt quality outcomes during the malting process has gained interest [18, 83]. Microbes are involved at every stage, from raw barley production to malting, and their functional diversity from seed exterior to interior may result either in beneficial or detrimental malt quality outcomes. To this end, we investigated the microbial community structure of malting barley seed endophytes to delineate the effects of genotype and location and the concomitant effects of their microbial composition on malt quality.

Plant genotypes has been implicated in microbial community structure, diversity, and assembly in different plant species, including *Medicago truncatula* [21], *Glycine max* [66], *Olea europaea* [70], wheat [109] and other cereals [69]. Our working hypothesis was that bacterial and fungal seed endophytic microbes have similar strategies for colonizing seed interior and hence face similar selection pressure, irrespective of barley seed genotypes. However, the observed results prove this not to be the case. We observed that not only genotype and location significantly influence the seed endophytic microbial communities, but their interaction effects were also significant. In fact, for some beta diversity estimates, the interaction (genotype x location) explained more variation in seed bacterial (Supplementary Table S5) and fungal microbial communities (Supplementary Table S6), than either genotype or location effects. These observations can be explained in part by the fact that seed metabolomes are unique to each genotype and their exudation may be modified by the environment to preferentially recruit certain beneficial microbes critical to plant fitness, especially when faced with onslaughts from biotic or abiotic stressors [67, 103]. In addition, the host defense system has been implicated in shaping seed endophytic microbial communities [42, 56] and as such, host genetic control of the defense responses may play a huge role in the recruitment or rejection of certain seed microbes. Alpha and beta diversity indices of barley genotypes across locations showed that AAC Synergy and ND Genesis have a similar pattern of distribution of bacterial species and abundance compared to Conlon (Fig. 1B and Fig. 3A-C). The very low variance in beta diversity indices (Table 3) between AAC Synergy and ND Genesis coupled with the few genera that were differentially abundant between these two genotypes (Supplementary Figure S11G) suggests similarities in strategies adopted by ND Genesis and AAC Synergy in recruiting and repelling bacterial species that shaped their microbiomes. Notably, a similar pattern was observed for ND Genesis and AAC Synergy fungal communities (Fig. 2B, Fig. 3D and Supplementary Figure S12G), albeit to a lesser extent. Also, we observed that barley samples from Crookston location consistently showed higher alpha diversity indices (Fig. 1B) and clustered separately (Fig. 3A, Supplementary Figure S2) from the rest of the samples. This observation further strengthens the pivotal roles of location and their interactions in modulating seed endophytic community diversity and assembly. These observations agree with other studies [53, 70, 76].

The taxonomic composition analysis indicated that the most abundant genera for bacterial and fungal communities were *Bacillus* (belonging to phylum Firmicutes) and *Blumeria* (belonging to phylum Ascomycota), respectively (Supplementary Figure S6 and Supplementary Figure S7). These observations were unexpected given that barley, sorghum, rice and wheat seed endophytic bacterial communities are mostly dominated by the Proteobacteria, with notable exceptions for coffee, soybeans, and *Brachypodium* seed microbiomes that are mostly dominated by the Firmicutes [52]. Bacterial species constituting the Firmicutes phylum found in C4 and CAM plants were associated with strong adaptation capacity, with Proteobacteria mostly prevalent in C3 seeds being replaced by the more resistant Firmicutes taxa in C4 and CAM photosynthetic groups [40]. *Bacillus subtilis* demonstrated significant control on wheat powdery mildew [113] while other *Bacillus* species, such as *Bacillus licheniformis* and *Bacillus* *amyloliquefaciens* serve as biological control agents (BCAs) to control fungal diseases in the field [22]. Pathogen-infected plants can specifically recruit and enrich beneficial microbial communities to act as antagonists to phytopathogens [13]. Based on these earlier observations, we speculate that the *Bacillus*-dominated bacterial seed endophytes maybe recruited to mitigate the effects of *Blumeria graminis*, a well-known obligate biotrophic fungus phytopathogen that causes powdery mildew impacting several economically important crops. We did not notice any discernable pattern in the abundance of *Blumeria* across genotypes and location except for ND Genesis in Casselton location which showed a slight decrease in the abundance based on centered log ratio estimates (Fig. 6E). We observed another core genus *Xanthomonas* that is abundant and commonly detected in all our genotype-location samples except for ND Genesis and AAC Synergy from St. Paul (Fig. 6B, Supplemental Table S2). *Xanthomonas* is a major group of pathogenic bacteria infecting major cereal grains like barley, although, non-pathogenic forms have also been detected [92]. To ascertain whether the genus *Xanthomonas* belonged to the pathogenic or non-pathogenic forms, we conducted a blastN analysis and found that the 260 bp sequence shared 100% homology with *Xanthomonas translucens* (Supplementary Figure S8), the causative pathogen for bacterial leaf streak disease (BLS) in cereals that affects grain filling. Although seeds are known to be the primary source of *X. translucens* pv. *translucens* inoculum [32], the prevalence and abundance of *Xanthomonas* as a core genus in barley seed endophytes was unexpected. We observed that ND Genesis and AAC Synergy from St. Paul were devoid of genus *Xanthomonas,* leading to hypothesize that their host defense system may play a central role in selectively repressing *Xanthomonas* (Fig. 6B and Supplementary Table S2). Interestingly, previous work suggested a key role for the host defense system in promoting microbiota homeostasis and mitigating potential invasion of opportunistic pathogens [63, 87]. Considering recent evidence that indicated a reemergence of *X. translucens* pv. *translucens* within the barley-growing regions of North America [16, 29, 46, 95, 102], possibly due to the large-scale long-distance dissemination of the pathogen through seed [32], disease-free seed production in certain geographical locales may be a viable option to mitigate the spread of BLS. Also worth mentioning is the appearance of a negative trend between *Bacillus* and *Xanthomonas* genera across genotype and location, as the abundance of genus *Xanthomonas* (for example in St Paul) lowered, abundance of genus *Bacillus* increased in Conlon and this trend was noticeably consistent across the samples (Fig. 6A and B).

Seeds harbor microbiota that can be transmitted to the plants that develop from them [14] and understanding how microbial diversity changes across genotypes and locations could be critical to plant fitness. In this study, we observed that a greater proportion of bacterial ASVs were shared across genotypes (Fig. 6C) and across location (Fig. 6D) while the greater proportion of the fungal ASVs were unique to each genotype (Fig. 6F) and location (Fig. 6G), indicating that the underlying mechanism that shapes the recruitment of bacterial and fungal seed endophytes differ potentially due to differences in selective pressure.

The microbiological status of the grain pre- and postharvest, and during different stages of malting could influence malt quality outcomes [18]. Association studies investigating the link between human gut microbiome and human personality traits [51], obesity [77], asthma [8], as well as mental health conditions [2] have led to investigations of plant microbial communities and economically important traits. In this study, association analysis suggests a negative correlation between bacterial alpha diversity indices (Faith’s PD and Shannon indices) and malt quality traits such as barley protein (BP), free amino nitrogen (FAN), diastatic power (DP) and alpha amylase (AA) (Fig. 4B-D and Supplementary Figure S4A and S4B). Given these observations, we investigated whether the nature of the relationship between Faith’s PD of St. Paul and Crookston location (Fig. 4A) would replicate the negative relationship between malt quality traits and Faith’s PD (for example, increase in Faith PD in Crookston would be associated with a decrease in BP, FAN, DP, and AA) when applied to a different malt quality dataset from these two locations. Surprisingly, the same pattern of negative correlation between Faith’s PD and some malt quality traits (Fig. 4F) were detected with significantly higher values observed for BP, DP, and FAN for St Paul samples (lower Faith’s PD) and significantly lower values for BP, DP, and FAN for Crookston samples (higher Faith’s PD). Based on these findings, we argue that increased bacterial abundance negatively influence malt quality traits. This idea is consistent with previous findings that excessive bacterial growth on barley grain could retard mash filtrations due to increased abundance of bacterial exopolysaccharides [44, 60] and due to microbial interference with respiration during the malting process [93].

Lower levels of β-D-glucan content and higher levels of β-glucanases in malted barley correlates with better malting performance, resulting in reduced viscosity and enhanced brewing efficiency and quality [43]. Regarding the nature of relationships between fungal communities and malt quality traits, we only observed a significant negative relationship between fungal abundance and β-D-glucan content (Fig. 5A and B). Previous work reported an up-regulation of β-glucanases in cereals infected with *Fusarium* spp. [10, 86], but it was unclear whether the elevated levels were derived solely from cereals or if they also originated from fungi (Geibinger et al., 2022). Enhanced activity of β−1,3-glucanases was reported in barley (*Hordeum vulgare* L.) after infection with *Blumeria graminis* (DC) Speer f. sp. *Hordei* [47]. Another study reported a decrease in β-glucan content after artificial inoculation with *Fusarium graminearum* in oats, linking this reduction to the demand for glucose units required for their nutrition and growth [45]. Also, leaf β-D-glucan content was significantly decreased in oat after artificial infection with *Blumeria graminis* [41], possibly due to the fact that β−1,3-glucanases of fungal origin exhibited similar mechanisms of action on cereal β-D-glucan [24, 25, 114]. Given the abundance of *Blumeria graminis* in this study (Fig. 2A), we hypothesize that the observed significant negative correlation between fungal abundance and beta-glucan content is mediated by activities of fungal β−1,3-glucanases hydrolyzing cereal β-D-glucan (Fig. 5A and B). It is noteworthy that the abundance of *Blumeria graminis* is not only related to its effects on beta glucan content, DP and AA are also significantly positively associated with this genus (Fig. 5C). The significant down-regulation of alpha amylase inhibitors was reported during grain development in *Blumeria graminis* infected wheat cultivar compared to uninfected controls [64]. It is however unclear whether a similar mechanism exist in barley infected with powdery mildew, given that similar down-regulation of the endogenous barley alpha amylase/subtilisin inhibitor (and subsequent increase in alpha amylase activity) has the potential to increase starch degradation and subsequently increase malt extract. Although, there was no significant correlation between genus *Blumeria* and malt extract, barley alpha amylase/subtilisin inhibitor against endogenous xylanase isozyme I (X-I) and alpha amylase isozyme II (AMY-II) activity in barley have been reported [101, 105].

Increase in *Fusarium* abundance has been implicated in beer gushing and grain quality deterioration [49, 108]. Our findings revealed that among the *Fusarium spp.* impacting malt quality, only *Fusarium poae* was identified and present at low levels (Supplementary Figure S7) and was not associated with any of the malt quality traits measured. It was not surprising to see a reduction in *Fusarium* abundance in our samples, given that the recommended malting cultivars (especially “malting check” cultivars) are known to undergo rigorous screening for low mycotoxin levels prior to their adoption for use by maltsters, who only purchase grain with < 0.5 ppm DON [112]. To this end, we speculate that a strong selection pressure against *Fusarium* may have favored the recruitment of other fungal pathogens, potentially affecting malt quality through their cell wall degrading enzymes.

Extractability is a key quality trait as malts with high extract are desired in the brewhouses. Bacterial genus like *Sphingomonas* produce gellan using starch as a substrate [36, 84] while *Pseudomonas* are known to produce a variety of different bacterial polysaccharides [9, 107]. Members of genus *Pantoea* are known for the production of exopolysaccharides and have been implicated in reducing wort filtration performance [60]. In this study, we observed that most of the bacterial genera were significantly negatively associated with malt extract except *Paenibacillus* that was significantly positively associated with malt extract (Fig. 4G). This observation may be partly attributed to the ability of *Paenibacillus* to produce significant amount of xylanase in malting milieu [71], with increased xylanase levels negatively correlated with wort viscosity and increased extract yield and brewing filterability [28]. Notably, our work demonstrated the significant abundance of *Paenibacillus* in certain genotypes and locations at the exclusion of others (for example, *Paenibacillus* is only present in Crookston and not St. Paul for all the genotypes; Supplementary Table S2 and Supplementary Table S7) and may influence malt quality traits.

### Future directions

Seed bio-priming is an economically viable and environmentally sustainable technique to stimulate the growth of beneficial microbes that enhance growth and induce disease resistance in crop plants. The exploration of the identified bacterial endophytes especially those in the genus Bacillus for biopriming will be a novel avenue worthy of further exploration for improving barley resiliency. In fact, based on this study, seed endophytes that inhibit the growth of pathogens such as the devastating scab disease caused by Fusarium has been reported recently [3]. Further studies on the negative interaction between Blumeria and Xanthamonas can be useful for mitigating the bacterial leaf streak resurgence in barley growing regions. Investigating the microbial shifts across different malting stages and their influence on malt quality traits (such as lowering beta glucan levels) provides an opportunity to improve the efficiency of the malting process. Microbiome analysis during malting stages (e.g. with seed samples from diverse geographical locations) can also be explored for the purpose of identifying beneficial microbes for manipulating the sugar profiles to cater to the needs of the brewing industry and the end consumer.

## Supplementary Information

Additional file 1. S1 Fig. S1. Rarefaction curves for malting barley seed endophytes across four locations. Rarefaction curves were made for all samples to evaluate the species richness, depth of sampling and sequencing coverage for bacterial 16S (A) and fungal ITS (B). S1 Fig. S2. - Bacterial community composition of malting barley seed endophytes across four locations. A-I represents the PCoA of 16S rRNA amplicon sequencing data across location for each cultivar based on unweighted unifrac (A-C), Bray-Curtis (D-F) and Jaccard distances (G-I). A,E,H are for ND Genesis; B,E,H are for AAC Synergy while C,E,H are for Conlon genotypes. S1 Fig. S3. Fungal community composition of malting barley seed endophytes across four locations. A-F represents the PCoA of fungal ITS sequencing data across location for each cultivar based on Bray-Curtis (A-C) and Jaccard distances (D-F). A,D are for ND Genesis; B, E are for AAC Synergy and C,F are for Conlon genotypes/cultivars. S1 Fig. S4. Negative relationship between Shannon index, bacterial genus taxa and some malt quality traits. For free amino nitrogen (FAN) (A), and alpha amylase (AA) (**B**) based on linear regression after adjusting for covariates genotype and location. S1 Fig. S5. UpSet plots of bacterial and fungal ASVs present in each location of Conlon, ND Genesis and AAC Synergy.(A-C) UpSet plot showing the number of bacterial ASVs that are shared between or are unique for all locations for Conlon (A), ND Genesis (B) and AAC Synergy (C). (D-F) UpSet plot showing the number of fungal ASVs that are shared between or are unique for all locations for Conlon (D), ND Genesis (E) and AAC Synergy (F). Number in color in A-F indicates shared ASVs common to all locations, while filled-in black dots with an edge between the dots indicates that these ASVs are present in multiple locations. S1 Fig. S6. Heatmap of the relative abundance of the top 30 bacterial seed endophytic communities of malting barley genotypes/cultivars across four locations. S1 Fig. S7. Heatmap of the relative abundance of the top 30 fungal seed endophytic communities of malting barley genotypes/cultivars across four locations. S1 Fig. S8. Feature ID sequence identification and BlastN analysis of 16S rRNA partial sequence of Xanthomonas. (A) Feature ID colored in orange represents genus Xanthomonas (B) BlastN analysis of the 260 bp sequence shared 100% homology with Xanthomonas translucens. S1 Fig. S9. Genotype and location pairwise comparison of the seed endophytic bacteria microbial taxonomic composition. For Crookston vs. St. Paul (A),  Carrington vs. Casselton (B), AAC Synergy vs. Conlon (C), Conlon vs. ND Genesis (D), and AAC Synergy and ND Genesis (E). For A-E, dot plots of the LDA scores (log 10) computed for features with differential abundance in each pairwise assessments as indicated above. S1 Fig. S10. Genotype and location pairwise comparison of the seed endophytic fungal microbial taxonomic composition. For Carrington vs. Casselton (A), Crookston vs. St. Paul (B), AAC Synergy vs. Conlon (C), Conlon vs. ND Genesis (D), and AAC Synergy and ND Genesis (E). For A-E, dot plots of the LDA scores (log 10) computed for features with differential abundance in each pairwise assessments as indicated above. S1 Fig. S11. Differential abundance analysis of seed endophytic bacterial communities of malting barley grown across four locations. (**A**) Pairwise differential abundance analysis (based on logFC) between St. Paul and Crookston locations at the genus level. Positive logFC indicates increased endophytic bacterial (EB) abundance in St Paul (decrease EB in Crookston), while negative logFC indicates decreased EB in St. Paul (increased EB in Crookston). (**B**) Pairwise differential abundance analysis (based on logFC) between Carrington and Casselton locations at the genus level. The negative logFC indicates decreased EB in Casselton (increased EB in Carrington). Only some genera are reported (for complete list see Table S7) (**C**). Venn diagram showing unique and shared ASVs between pairwise location comparisons (Complete list is available in Table S7). (**D -F**) Pairwise differential abundance analysis (based on logFC) between Conlon and AAC Synergy (**D**), Conlon and ND Genesis (**E**) and AAC Synergy and ND Genesis (**F**) at the genus level. For **D**, Positive logFC indicates increased EB abundance in Conlon (decrease EB in AAC Synergy), while negative logFC indicates decreased EB in Conlon (increased EB in AAC Synergy). For **E**, Positive logFC indicates increased EB abundance in Conlon (decrease EB in ND Genesis), while negative logFC indicates decreased EB in Conlon (increased EB in ND Genesis). For **F**, Positive logFC indicates increased EB abundance in AAC Synergy (decrease EB in ND Genesis), while negative logFC indicates decreased EB in AAC Synergy (increased EB in ND Genesis). (**G**) Venn diagram showing unique and shared ASVs between pairwise genotype comparisons (Complete list is available in Table S7). S1 Fig. S12. Differential abundance analysis of seed endophytic fungal communities of malting barley grown across four locations. (**A**) Pairwise differential abundance analysis (based on logFC) between Carrington and Casselton locations at the genus level. Positive logFC indicates increased endophytic bacterial (EB) abundance in Carrington (decrease EB in Casselton), while negative logFC indicates decreased EB in Carrington (increased EB in Casselton). (**B**) Pairwise differential abundance analysis (based on logFC) between St. Paul and Crookston locations at the genus level. Positive logFC indicates increased endophytic bacterial (EB) abundance in St. Paul (decrease EB in Crookston), while negative logFC indicates decreased EB in St. Paul (increased EB in Crookston). (**C**) Venn diagram showing unique and shared ASVs between pairwise location comparisons (Complete list is available in Table S8). (**D -F**) Pairwise differential abundance analysis (based on logFC) between Conlon and AAC Synergy (**D**), ND Genesis and Conlon (**E**) and ND Genesis and AAC Synergy (**F**) at the genus level. For **D**, Positive logFC indicates increased EB abundance in Conlon (decrease EB in AAC Synergy), while negative logFC indicates decreased EB in Conlon (increased EB in AAC Synergy). For **E**, Positive logFC indicates increased EB abundance in ND Genesis (decrease EB in Conlon), while negative logFC indicates decreased EB in ND Genesis (increased EB in Conlon). For **F**, Positive logFC indicates increased EB abundance in ND Genesis (decrease EB in AAC Synergy), while negative logFC indicates decreased EB in ND Genesis (increased EB in AAC Synergy). (**G**) Venn diagram showing unique and shared ASVs between pairwise genotypes comparisons (Complete list is available in Table S8).

Additional file 2.

## Data Availability

The bacterial and fungal sequence data generated in this study using NextSeq2000 have been deposited and are available in the NCBI Sequence Read Archive (SRA) under Bioproject PRJNA1108745.
