# Supplementary material for: Seed endophytes of malting barley from different locations are shaped differently and are associated with malt quality traits
Source: BMC Plant Biol. 2025 Feb 5;25:151. doi: 10.1186/s12870-025-06089-6 (PMC11796131; doi:10.1186/s12870-025-06089-6)
Supplement: Supplementary file 1 — Additional file 1. S1 Fig. S1. Rarefaction curves for malting barley seed endophytes across four locations. Rarefaction curves were made for all samples to evaluate the species richness, depth of sampling and sequencing coverage for bacterial 16S (A) and fungal ITS (B). S1 Fig. S2. - Bacterial community composition of malting barley seed endophytes across four locations. A-I represents the PCoA of 16S rRNA amplicon sequencing data across location for each cultivar based on unweighted unifrac (A-C), Bray-Curtis (D-F) and Jaccard distances (G-I). A,E,H are for ND Genesis; B,E,H are for AAC Synergy while C,E,H are for Conlon genotypes. S1 Fig. S3. Fungal community composition of malting barley seed endophytes across four locations. A-F represents the PCoA of fungal ITS sequencing data across location for each cultivar based on Bray-Curtis (A-C) and Jaccard distances (D-F). A,D are for ND Genesis; B, E are for AAC Synergy and C,F are for Conlon genotypes/cultivars. S1 Fig. S4. Negative relationship between Shannon index, bacterial genus taxa and some malt quality traits. For free amino nitrogen (FAN) (A), and alpha amylase (AA) (B) based on linear regression after adjusting for covariates genotype and location. S1 Fig. S5. UpSet plots of bacterial and fungal ASVs present in each location of Conlon, ND Genesis and AAC Synergy.(A-C) UpSet plot showing the number of bacterial ASVs that are shared between or are unique for all locations for Conlon (A), ND Genesis (B) and AAC Synergy (C). (D-F) UpSet plot showing the number of fungal ASVs that are shared between or are unique for all locations for Conlon (D), ND Genesis (E) and AAC Synergy (F). Number in color in A-F indicates shared ASVs common to all locations, while filled-in black dots with an edge between the dots indicates that these ASVs are present in multiple locations. S1 Fig. S6. Heatmap of the relative abundance of the top 30 bacterial seed endophytic communities of malting barley genotypes/cultivars across four [file 12870_2025_6089_MOESM1_ESM.pdf]

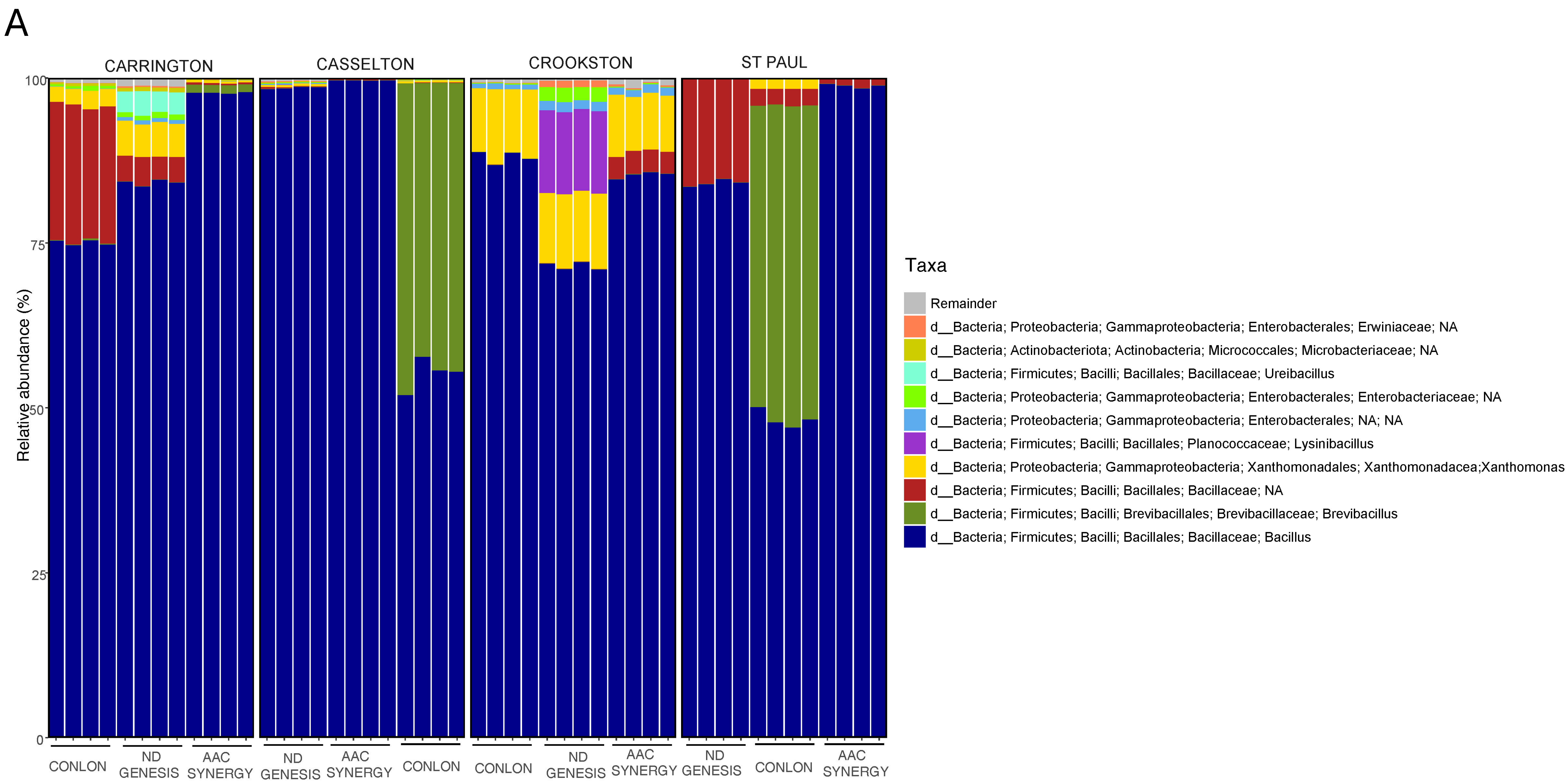
A
CARRINGTON
CASSELTON
CROOKSTON
ST PAUL
Relative abundance (%)
100
75
50
25
0
CONLON
ND GENESIS
AAC SYNERGY
ND GENESIS
AAC SYNERGY
CONLON
CONLON
ND GENESIS
AAC SYNERGY
ND GENESIS
CONLON
AAC SYNERGY
Taxa
Remainder
d__Bacteria; Proteobacteria; Gammaproteobacteria; Enterobacterales; Erwiniaceae; NA
d__Bacteria; Actinobacteriota; Actinobacteria; Micrococcales; Microbacteriaceae; NA
d__Bacteria; Firmicutes; Bacilli; Bacillales; Bacillaceae; Ureibacillus
d__Bacteria; Proteobacteria; Gammaproteobacteria; Enterobacterales; Enterobacteriaceae; NA
d__Bacteria; Proteobacteria; Gammaproteobacteria; Enterobacterales; NA; NA
d__Bacteria; Firmicutes; Bacilli; Bacillales; Planococcaceae; Lysinibacillus
d__Bacteria; Proteobacteria; Gammaproteobacteria; Xanthomonadales; Xanthomonadacea;Xanthomonas
d__Bacteria; Firmicutes; Bacilli; Bacillales; Bacillaceae; NA
d__Bacteria; Firmicutes; Bacilli; Brevibacillales; Brevibacillaceae; Brevibacillus
d__Bacteria; Firmicutes; Bacilli; Bacillales; Bacillaceae; Bacillus

B

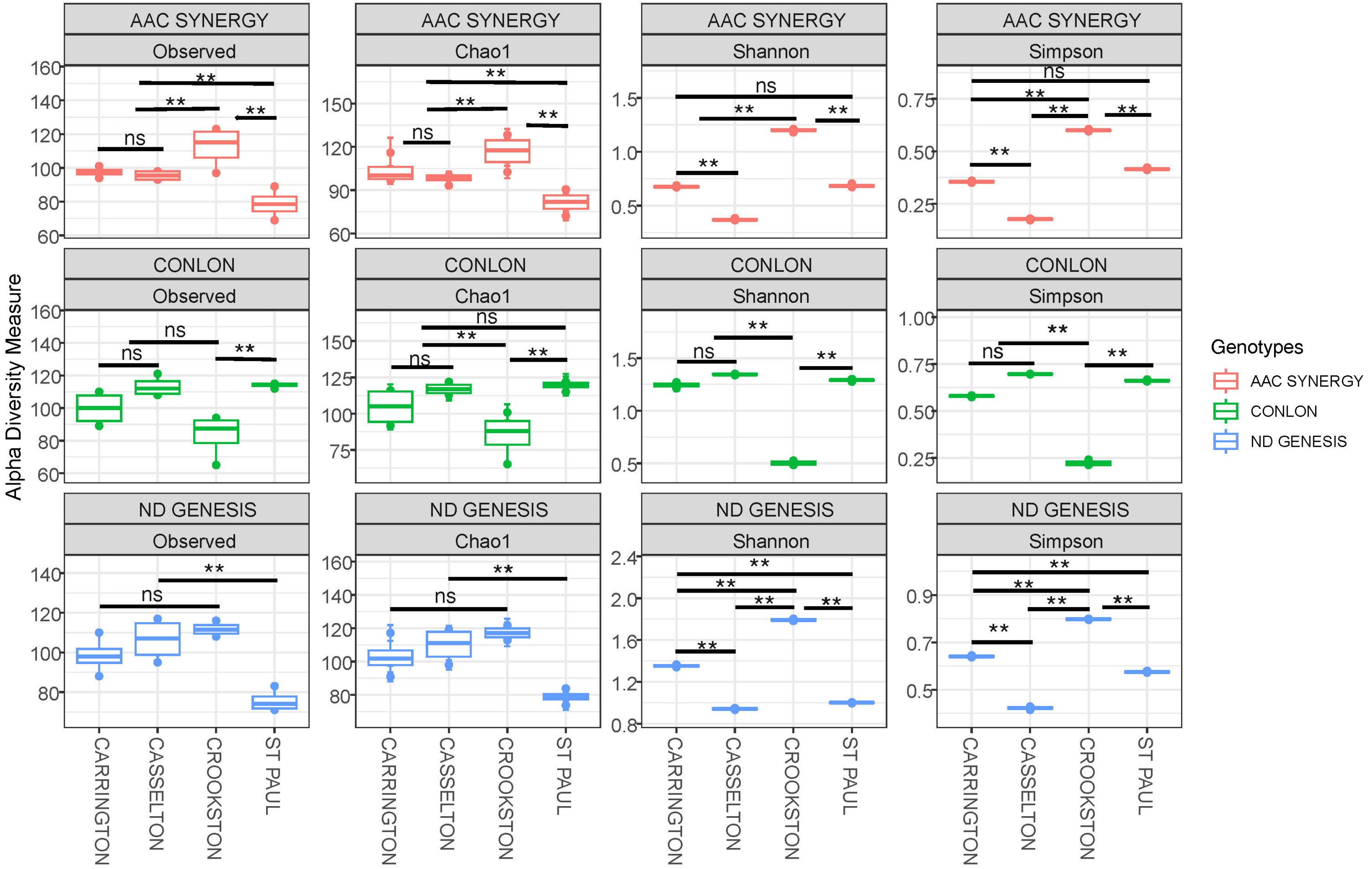

Figure 1. Taxonomic summary and bacterial diversity analyses of seed endophytes of three malting genotype/cultivars grown across four locations. (A) Stacked bar chart of the taxonomic composition of bacterial communities of different genotype-location sample types aggregated at the genus level. Each stacked column represents an independent sample (n=48). Only the top 10 most abundant taxa were colored individually, all the rest are colored in gray and listed as "Remainder". Samples are clustered by cultivar types (Conlon, ND Genesis and AAC Synergy) and location (Carrington, Casselton, Crookston and St Paul); **(B)** The bacterial seed endophytes alpha-diversity indices based on observed species richness, Chao1, Shannon diversity index, and Simpson varied for each malting cultivar/genotype across locations (ANOVA for all the alpha diversity indices were $p \leq 0.01$). Significant asterisks '**' ($p \leq 0.01$), ns = non-significant. Actual values of alpha-diversity indices can be seen in Table 1.

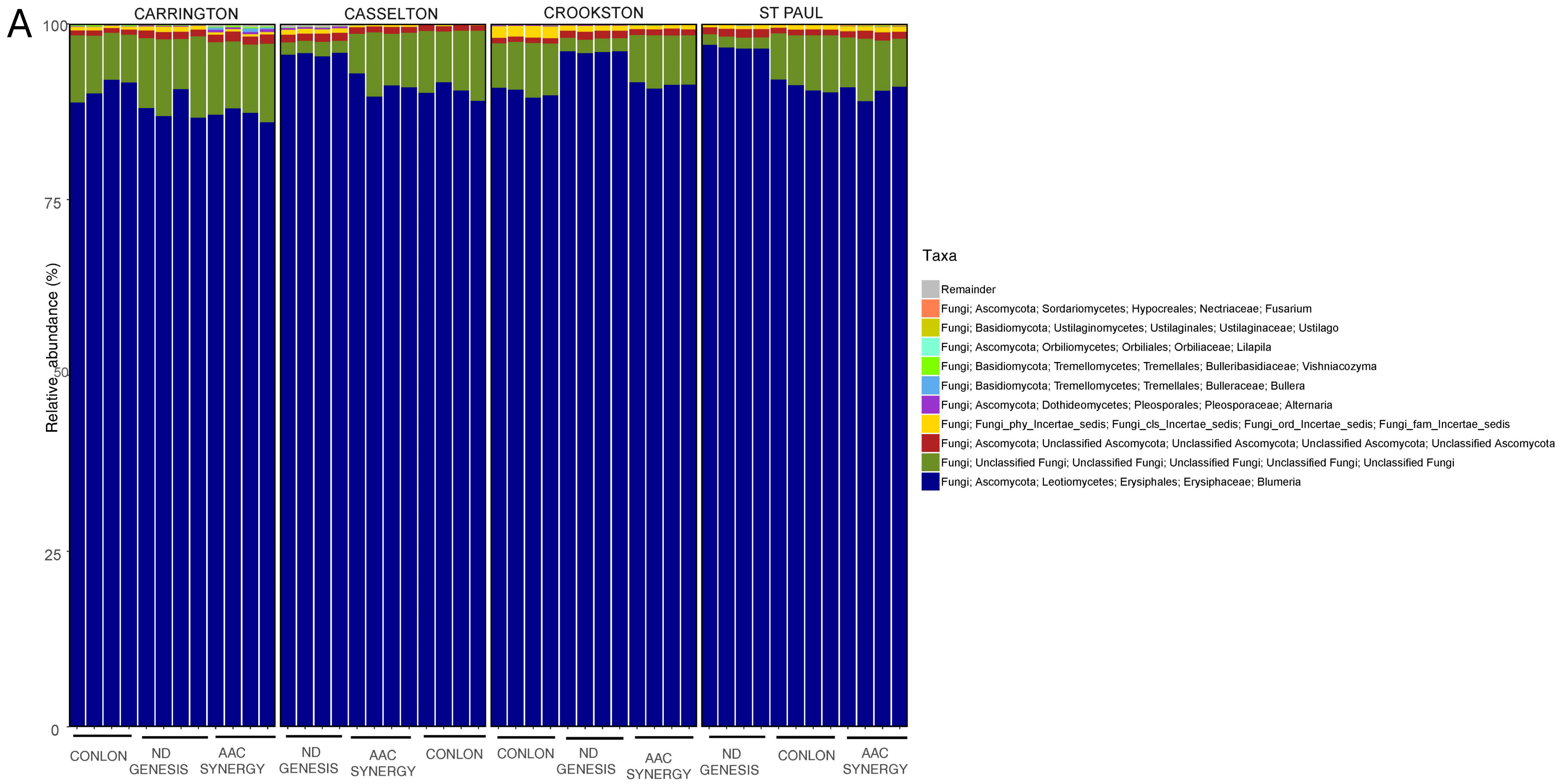
A
CARRINGTON
CASSELTON
CROOKSTON
ST PAUL
100
75
50
25
0
Relative abundance (%)
CONLON
ND GENESIS
AAC SYNERGY
ND GENESIS
AAC SYNERGY
CONLON
CONLON
ND GENESIS
AAC SYNERGY
ND GENESIS
CONLON
AAC SYNERGY
Taxa
Remainder
Fungi; Ascomycota; Sordariomycetes; Hypocreales; Nectriaceae; Fusarium
Fungi; Basidiomycota; Ustilaginomycetes; Ustilaginales; Ustilaginaceae; Ustilago
Fungi; Ascomycota; Orbiliomycetes; Orbiliales; Orbiliaceae; Lilapila
Fungi; Basidiomycota; Tremellomycetes; Tremellales; Bulleribasidiaceae; Vishniacozyma
Fungi; Basidiomycota; Tremellomycetes; Tremellales; Bulleraceae; Bullera
Fungi; Ascomycota; Dothideomycetes; Pleosporales; Pleosporaceae; Alternaria
Fungi; Fungi_phy_Incertae_sedis; Fungi_cls_Incertae_sedis; Fungi_ord_Incertae_sedis; Fungi_fam_Incertae_sedis
Fungi; Ascomycota; Unclassified Ascomycota; Unclassified Ascomycota; Unclassified Ascomycota; Unclassified Ascomycota
Fungi; Unclassified Fungi; Unclassified Fungi; Unclassified Fungi; Unclassified Fungi; Unclassified Fungi
Fungi; Ascomycota; Leotiomycetes; Erysiphales; Erysiphaceae; Blumeria

B

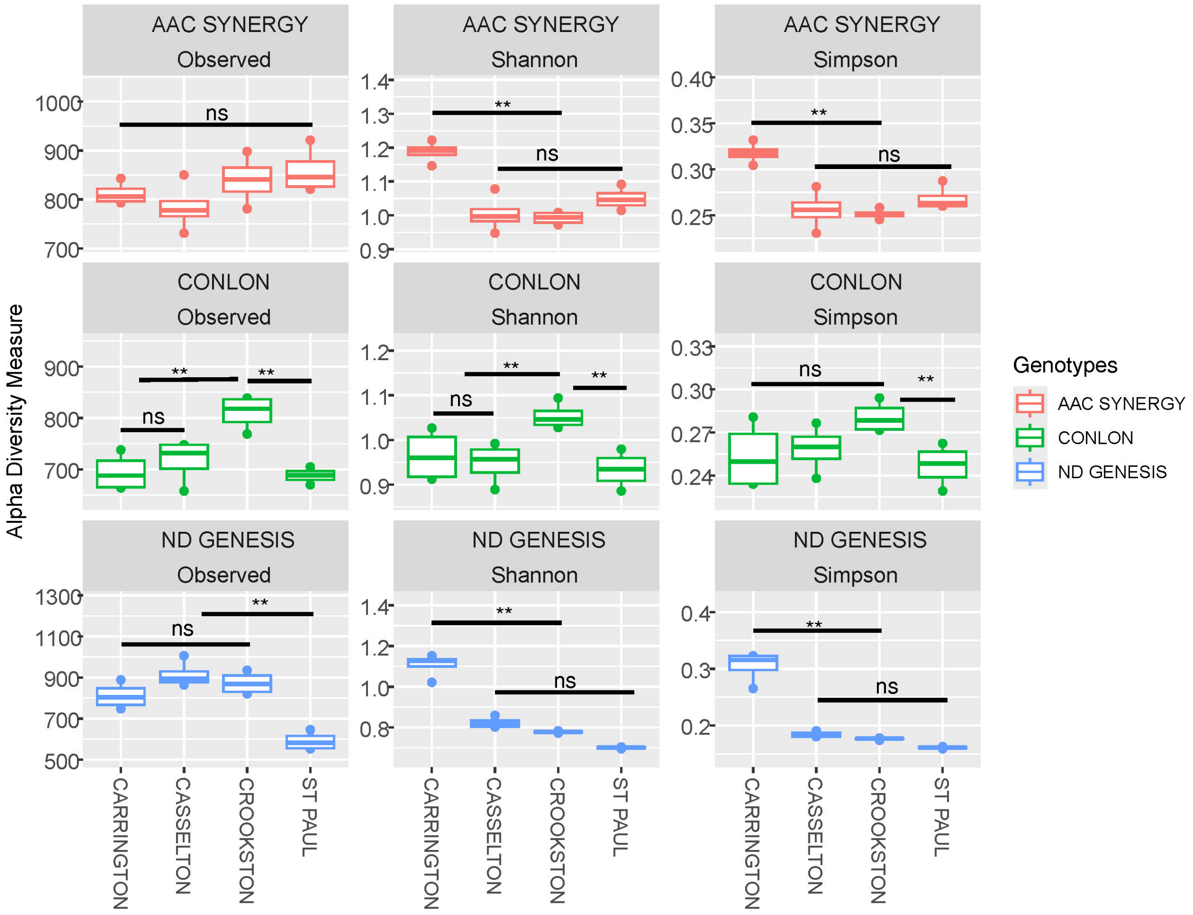

Figure 2. Taxonomic summary and fungal diversity analyses of seed endophytes of three malting barley genotype/cultivars grown across four locations. **(A)** Stacked bar chart of the taxonomic composition of fungi communities of different genotype-location sample types aggregated at the genus level. Each stacked column represents an independent sample (n=48). Only the top 10 most abundant taxa were colored individually, all the rest are colored in gray and listed as "Remainder". Samples are clustered by cultivar types (Conlon, ND Genesis and AAC Synergy) and location (Carrington, Casselton, Crookston and St Paul); **(B)** The fungal seed endophytes alpha-diversity indices based on observed species richness, Shannon diversity index, and Simpson varied for each malting cultivar/genotype across locations (ANOVA for all the alpha diversity indices were significant). Significant asterisks '**' ($p \leq 0.01$), ns = non-significant. Actual values of alpha-diversity indices can be seen in Table 2.

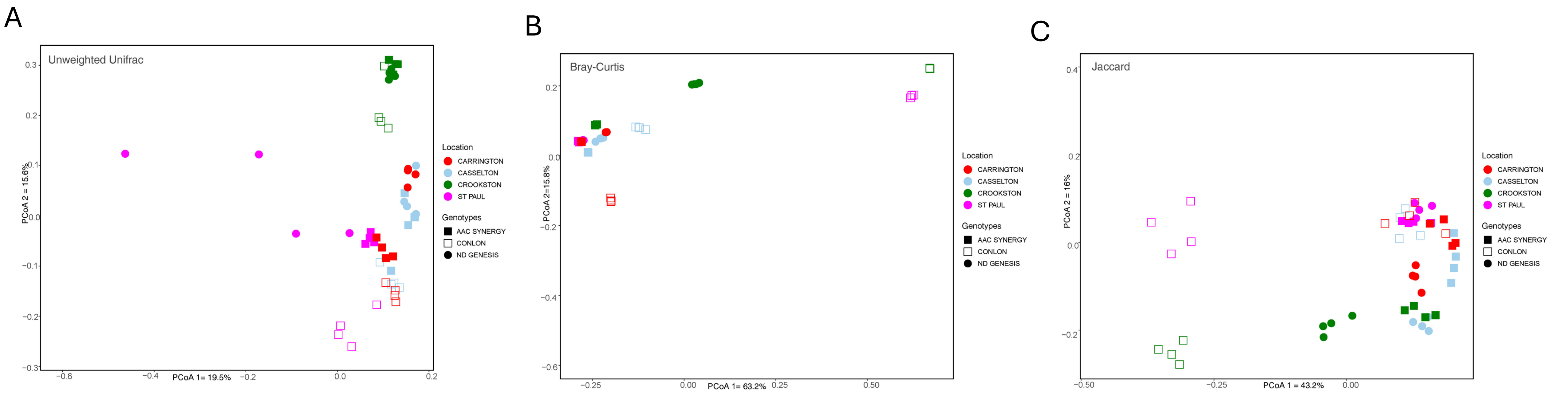

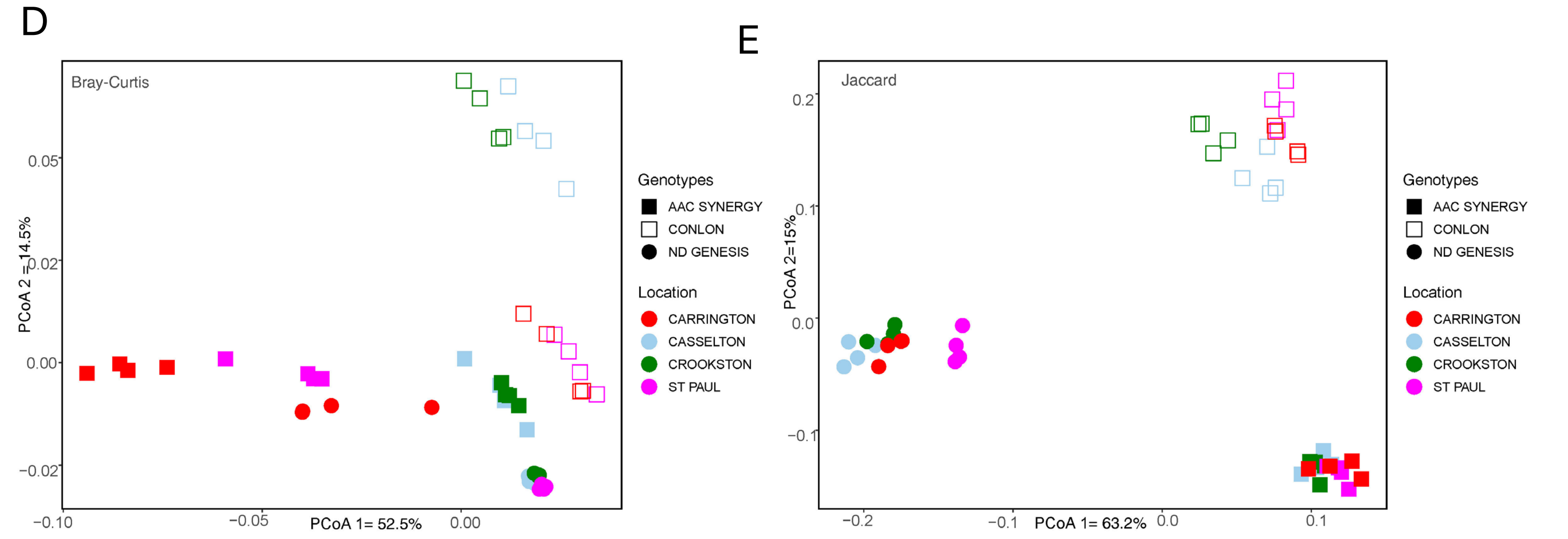

Figure 3. Bacterial and fungal communities of malting barley seed endophytes in relation to cultivar and location. A-C represents the PCoA of 16S rRNA amplicon sequencing data while **D** and **E** represents PCoA of ITS amplicon sequencing data of genotype-location samples based on unweighted unifrac (**A**), Bray-Curtis (**B**, **D**) and Jaccard distances (**C**, **E**). Each shape and color represent one replicate sample for genotype and location respectively for bacterial and fungal communities. Adonis in vegan package in R verified significant differences between the genotypes and locations ($p \leq 0.001$). Pairwise PERMANOVA estimates are presented in Table 3 (16s) and 4 (ITS).

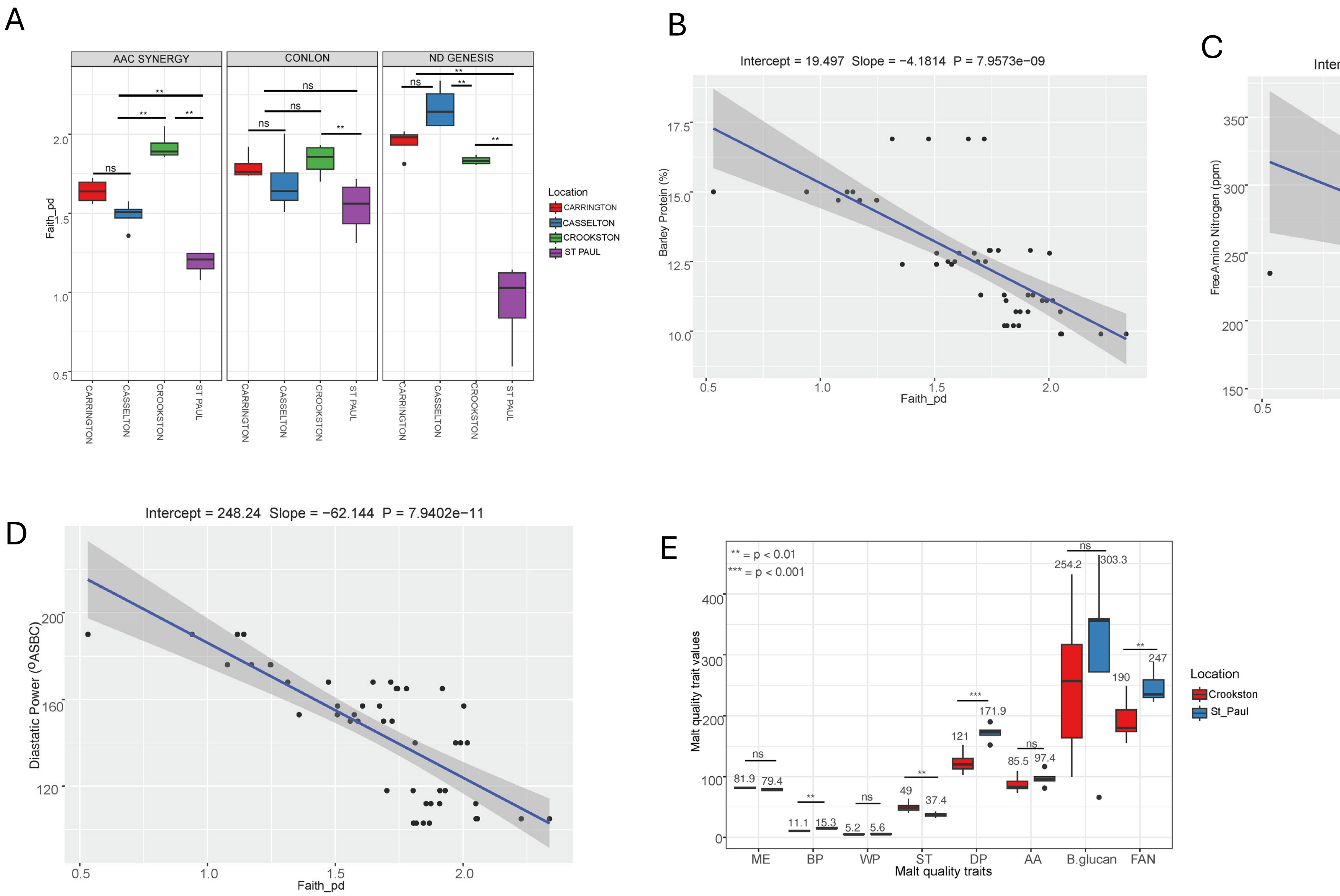
A
AAC SYNERGY
CONLON
ND GENESIS
Faith_pd
ns
**
ns
ns
ns
**
ns
**
**
**
CARRINGTON
CASSELTON
CROOKSTON
ST PAUL
Location
CARRINGTON
CASSELTON
CROOKSTON
ST PAUL
B
Intercept = 19.497 Slope = −4.1814 P = 7.9573e−09
Barley Protein (%)
Faith_pd
C
Intercept = 355.4 Slope = −72.241 P = 0.001798
FreeAmino Nitrogen (ppm)
Faith_pd
D
Intercept = 248.24 Slope = −62.144 P = 7.9402e−11
Diastatic Power (°ASBC)
Faith_pd
E
** = p < 0.01
*** = p < 0.001
Malt quality trait values
Malt quality traits
ME
BP
WP
ST
DP
AA
B.glucan
FAN
81.9
79.4
11.1
15.3
5.2
5.6
49
37.4
121
171.9
85.5
97.4
254.2
303.3
190
247
Location
Crookston
St_Paul

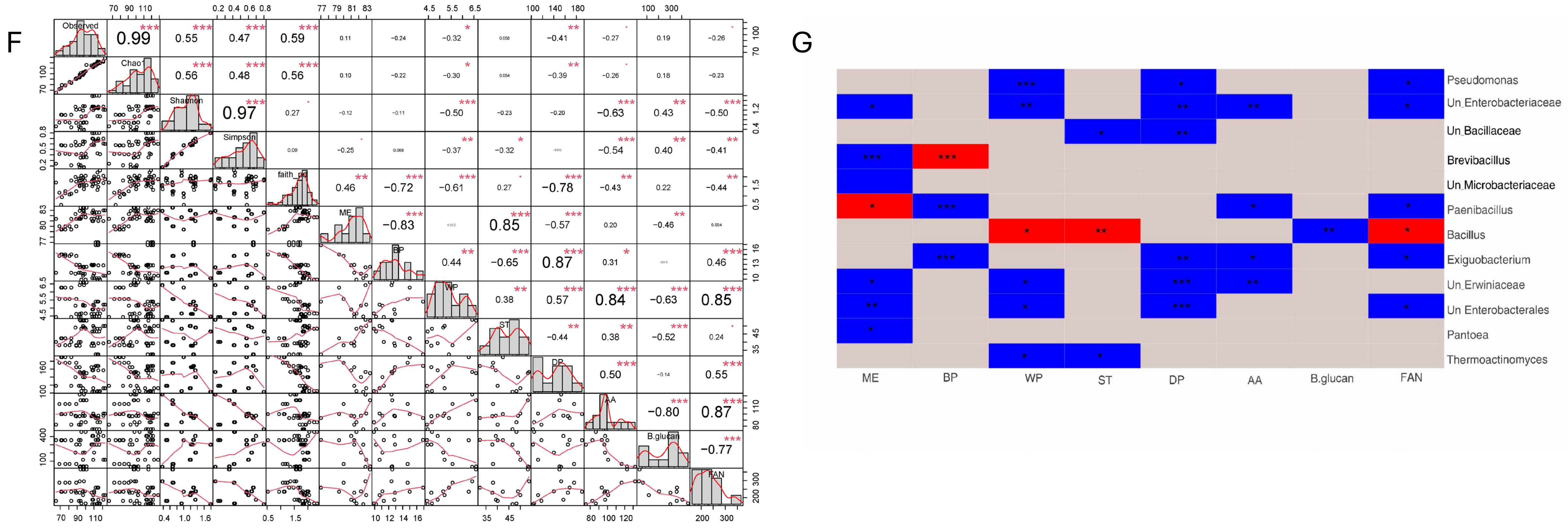

Figure 4. Association analysis between bacterial alpha diversity, along with bacterial genus taxa, and malt quality traits. (**A**) Alpha diversity metric (Faith_pd) of bacterial seed endophytes of three malting barley genotype/cultivars grown across four locations. (**B-D**) Negative relationship between Faith_pd and BP, barley protein (**B**), free amino nitrogen (FAN) (**C**), diastatic power (DP) (**D**) based on linear regression after adjusting for covariates genotype and location; (**E**) Malt quality analyses of Conlon, ND Genesis and AAC Synergy from Crookston and St. Paul that were statistically assessed using Welch test (N = 24, 15 samples from Crookston and 9 samples from St. Paul). (**F**) Correlation analysis between alpha diversity metrics and malt quality traits. (G) Association analyses between bacterial taxa (genus level) and malt quality traits after adjusting for genotype and location effects in a linear mixed model. Only genera significantly associated with malt quality traits were shown. Red color indicates significant positive association; blue color indicates significant negative association; grey color indicates no association. Significant asterisks '*' indicates p ≤ 0.05; '**' indicates p ≤ 0.01; '***' indicates p ≤ 0.001. Un = Unclassified.

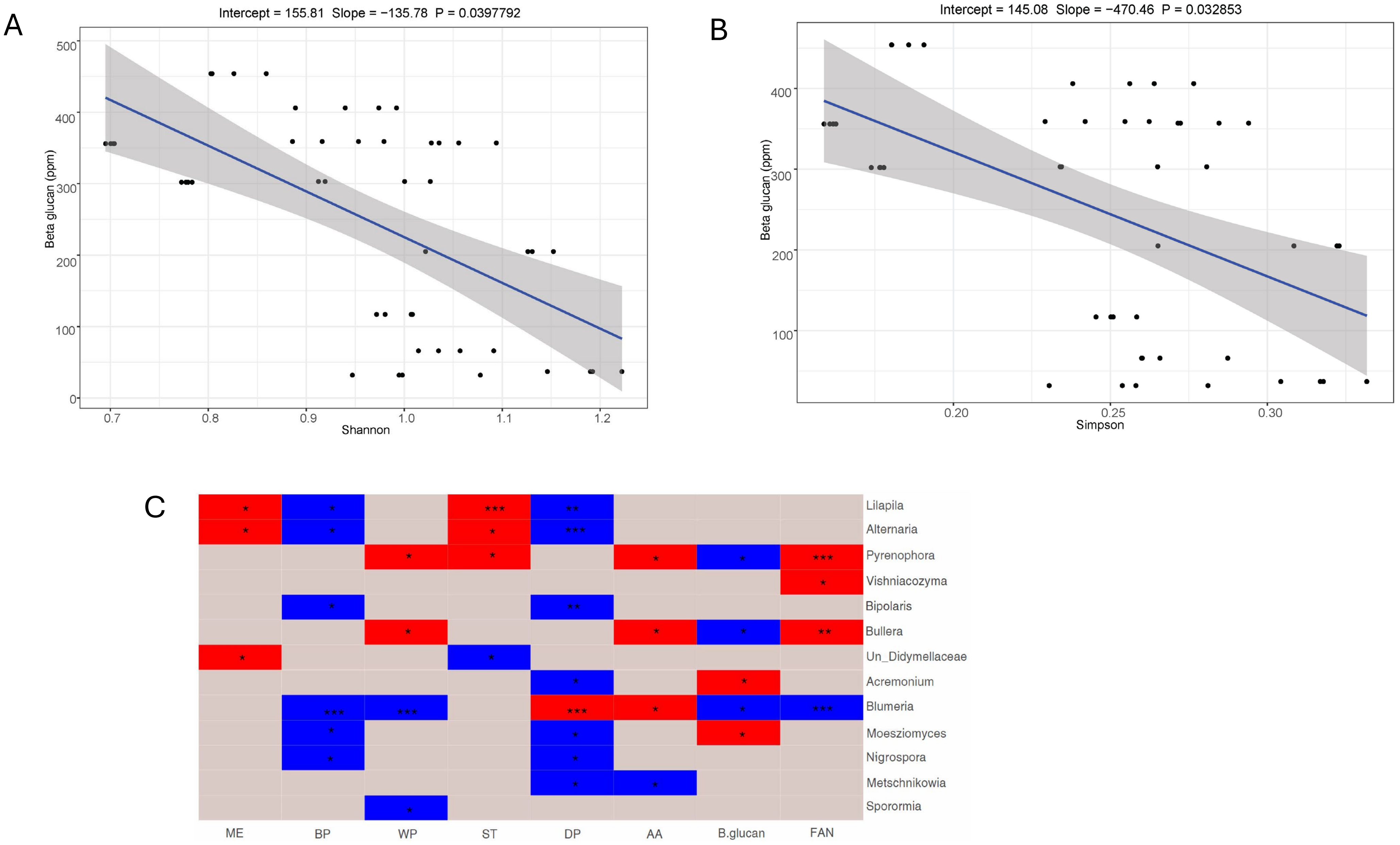
A
Intercept = 155.81 Slope = −135.78 P = 0.0397792
Beta glucan (ppm)
Shannon
B
Intercept = 145.08 Slope = −470.46 P = 0.032853
Beta glucan (ppm)
Simpson
C
Lilapila
Alternaria
Pyrenophora
Vishniacozyma
Bipolaris
Bullera
Un_Didymellaceae
Acremonium
Blumeria
Moesziomyces
Nigrospora
Metschnikowia
Sporormia
ME
BP
WP
ST
DP
AA
B.glucan
FAN

D

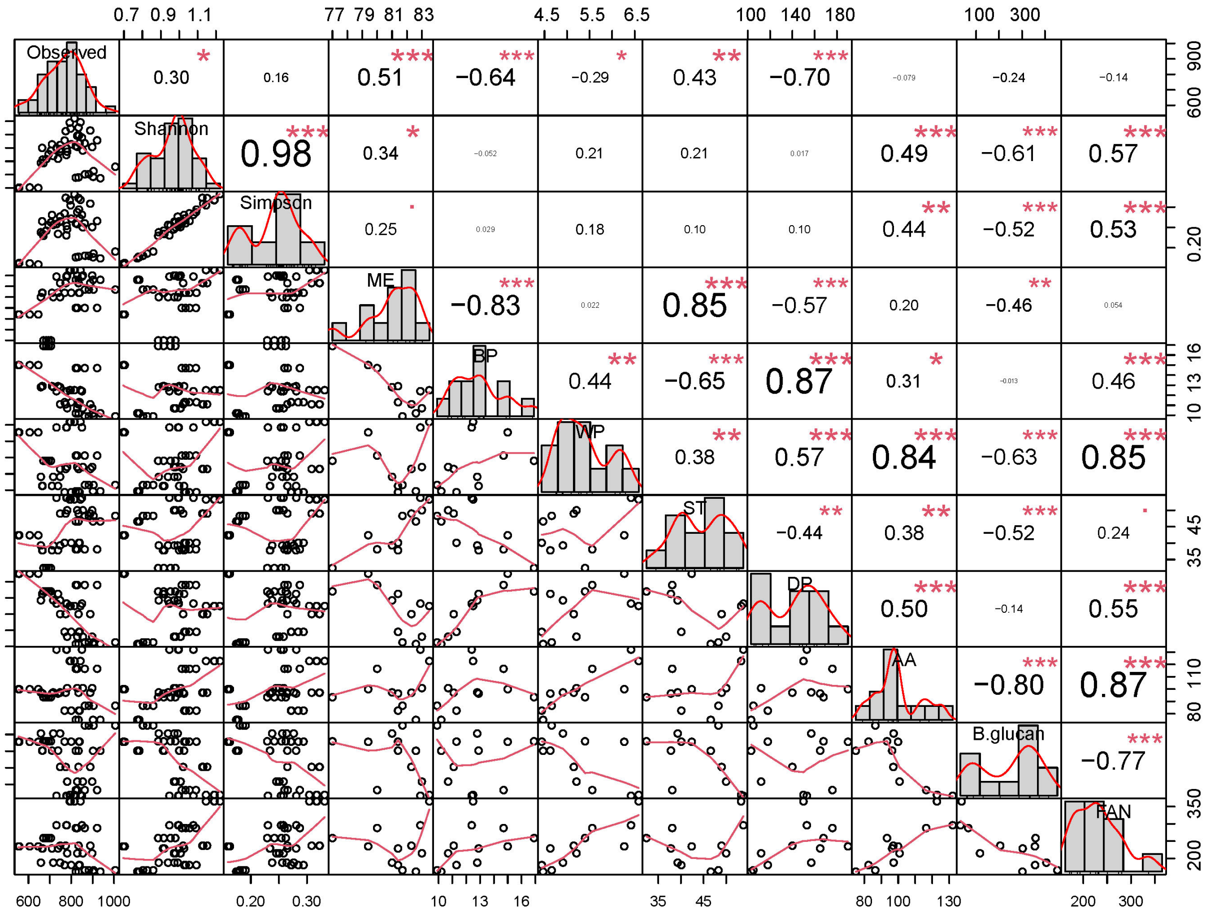

Figure 5. Association analysis between fungal alpha diversity, along with fungal genus taxa and malt quality traits. Shannon index (**A**) and Simpson index (**B**) and their negative association with beta glucan after adjusting for covariates genotype and location effects. (**C**) Relationship between fungal taxa (genus level) and malt quality traits after adjusting for genotype and location effects in a linear mixed model. Only genus significantly associated with malt quality traits were shown. Red color indicates significant positive association; blue color indicates significant negative association; grey color indicates no association. (**D**) Correlation analysis between alpha diversity metrics and malt quality traits. Significant asterisks '*' indicates p ≤ 0.05; '**' indicates p ≤ 0.01; '***' indicates p ≤ 0.001. Un = Unclassified.

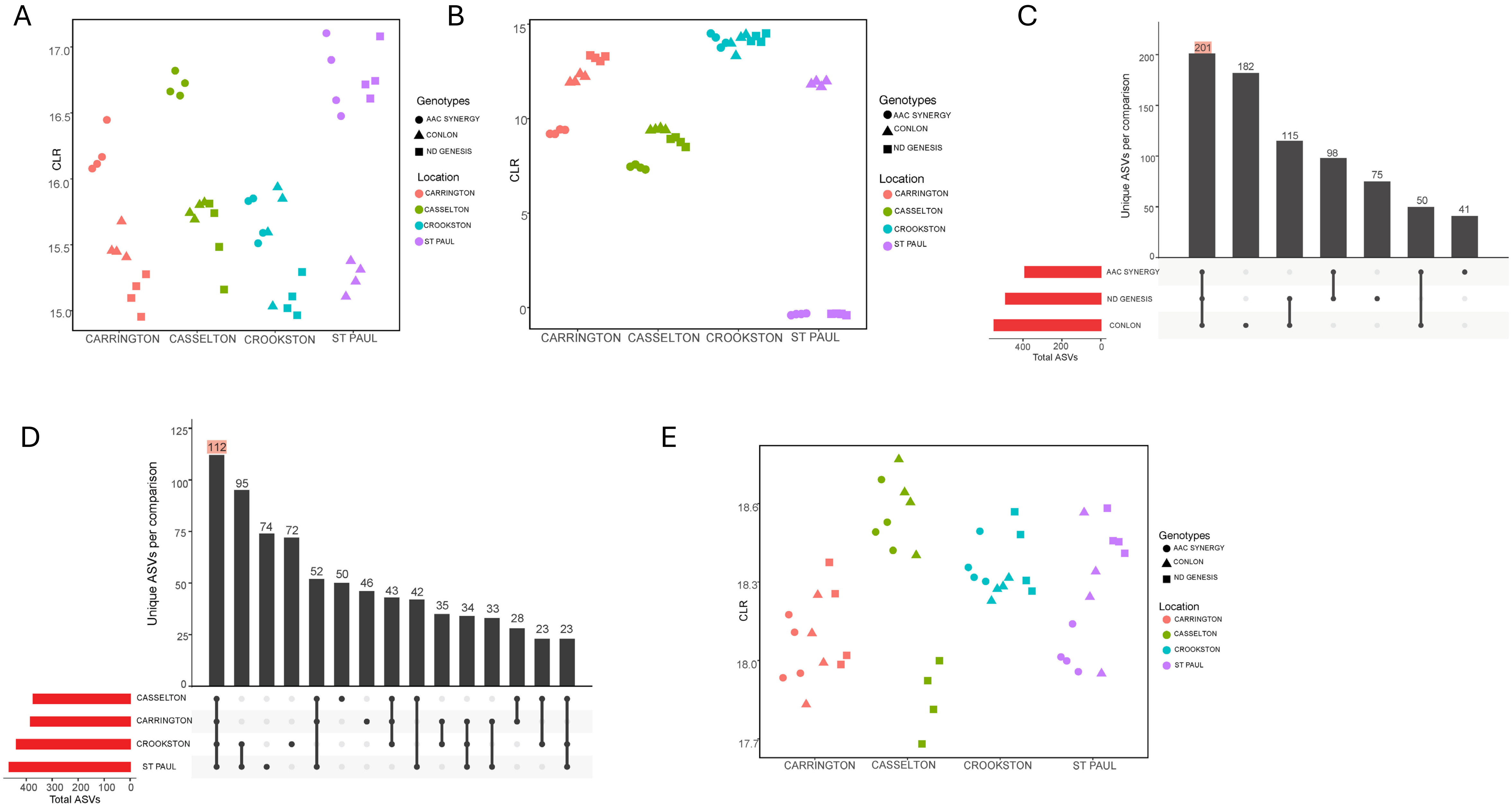
A
CLR
17.0
16.5
16.0
15.5
15.0
CARRINGTON
CASSELTON
CROOKSTON
ST PAUL
Genotypes
AAC SYNERGY
CONLON
ND GENESIS
Location
CARRINGTON
CASSELTON
CROOKSTON
ST PAUL
B
CLR
15
10
5
0
CARRINGTON
CASSELTON
CROOKSTON
ST PAUL
Genotypes
AAC SYNERGY
CONLON
ND GENESIS
Location
CARRINGTON
CASSELTON
CROOKSTON
ST PAUL
C
Unique ASVs per comparison
200
150
100
50
0
201
182
115
98
75
50
41
AAC SYNERGY
ND GENESIS
CONLON
400
200
0
Total ASVs
D
Unique ASVs per comparison
125
100
75
50
25
0
112
95
74
72
52
50
46
43
42
35
34
33
28
23
23
CASSELTON
CARRINGTON
CROOKSTON
ST PAUL
400
300
200
100
0
Total ASVs
E
CLR
18.6
18.3
18.0
17.7
CARRINGTON
CASSELTON
CROOKSTON
ST PAUL
Genotypes
AAC SYNERGY
CONLON
ND GENESIS
Location
CARRINGTON
CASSELTON
CROOKSTON
ST PAUL

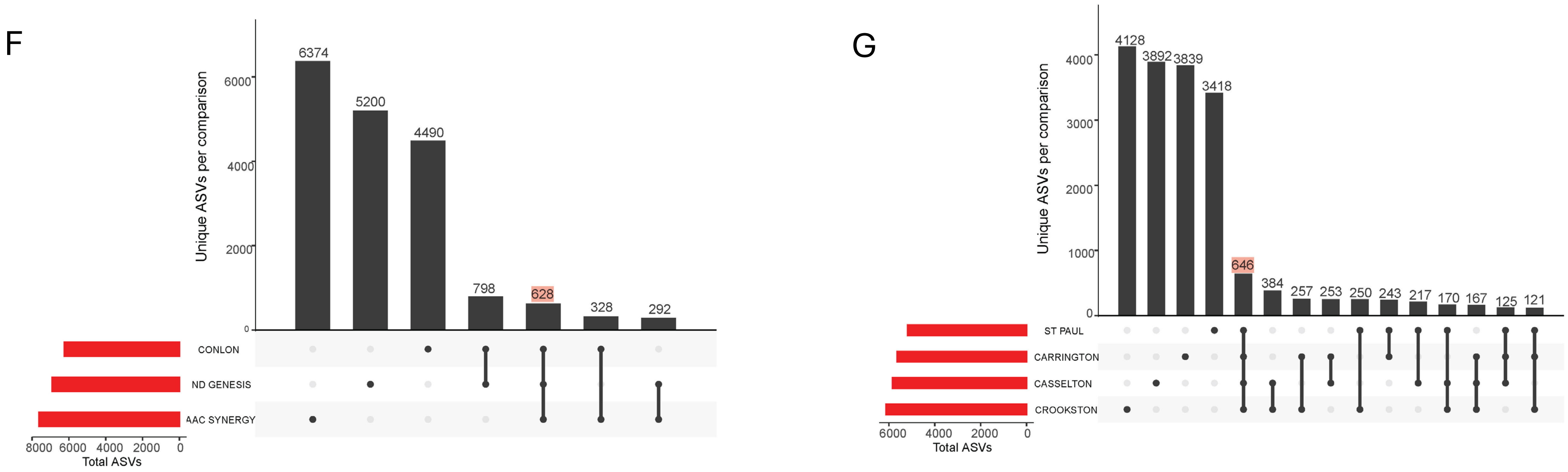

Figure 6. Abundance and UpSet plots of bacterial and fungal seed endophytes in relation to cultivar and location. Centered log ratio of ASV abundances for genus Bacillus (**A**) and Xanthomonas (**B**) among cultivars across location. (**C**) UpSet plot showing the number of bacterial ASVs that are shared between or are unique for AAC Synergy, ND Genesis and Conlon. (**D**) UpSet plot showing the number of bacterial ASVs that are shared between or are unique for Casselton, Carrington, Crookston and St Paul. (**E**) Centered log ratio of ASV abundances for genus Blumeria among cultivars across location. (**F**) UpSet plot showing the number of fungal ASVs that are shared between or are unique for AAC Synergy, ND Genesis and Conlon. (**G**) UpSet plot showing the number of fungal ASVs that are shared between or are unique for Casselton, Carrington, Crookston and St Paul. For **C, D, F, G**, number in color indicates number of shared ASVs common across all cultivar or location.

Supplementary Figures

Seed endophytes of malting barley from different locations are shaped differently and are associated with malt quality traits

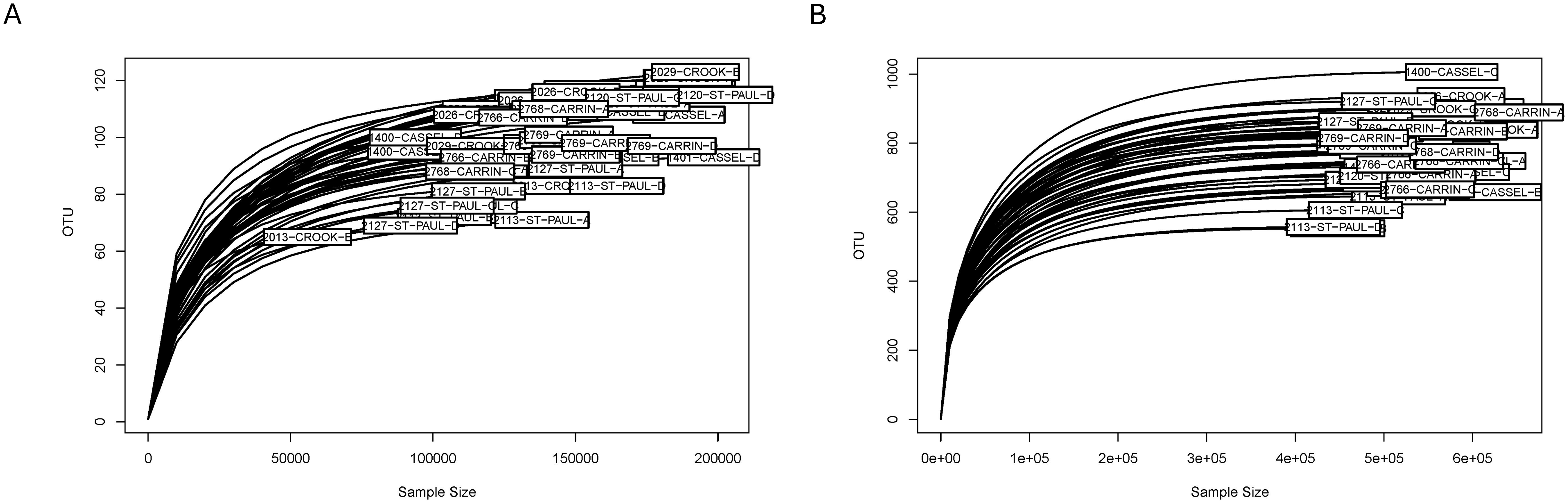

S1 Fig. S1. Rarefaction curves for malting barley seed endophytes across four locations. Rarefaction curves were made for all samples to evaluate the species richness, depth of sampling and sequencing coverage for bacterial 16S (A) and fungal ITS (B).

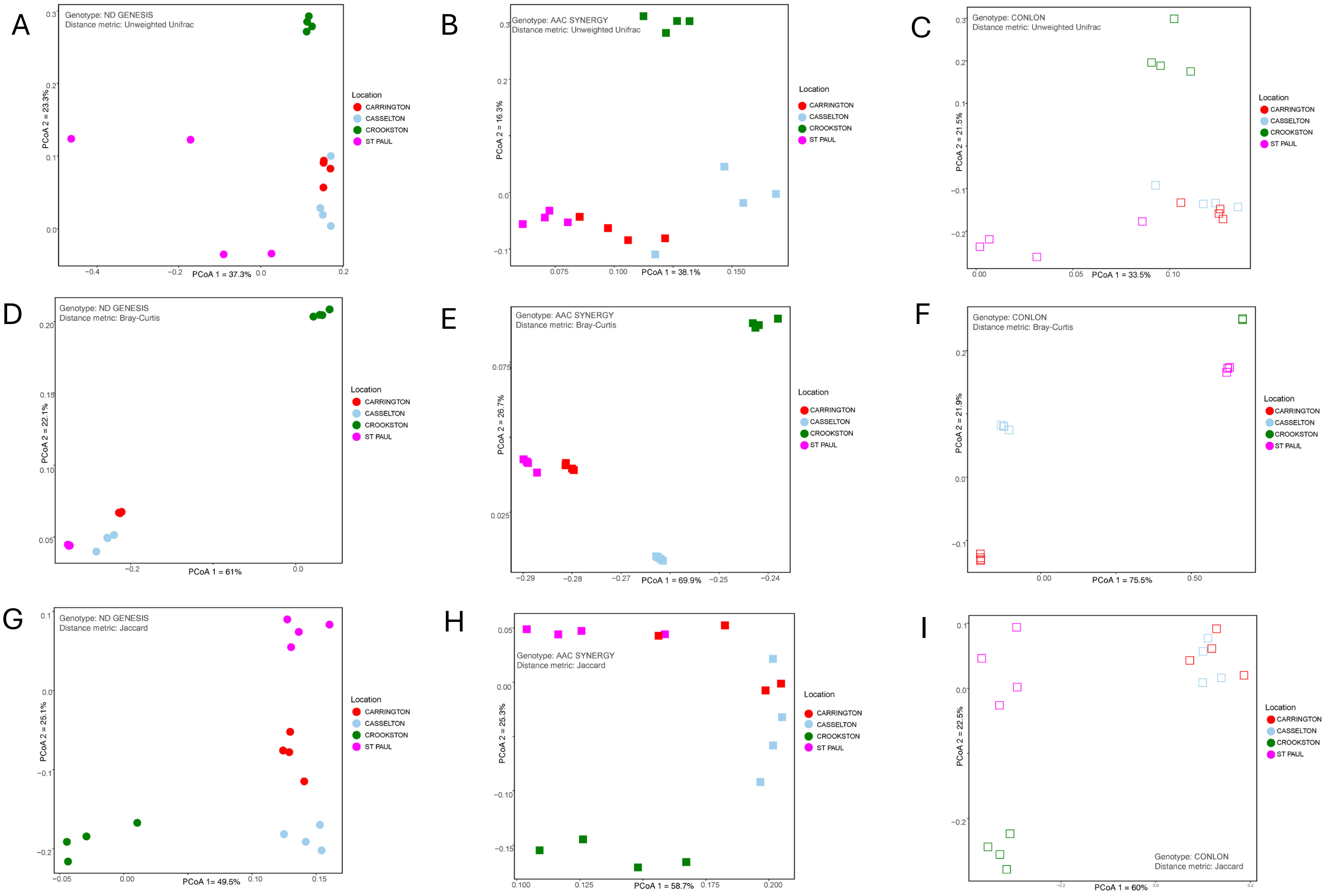

S1 Fig. S2. - Bacterial community composition of malting barley seed endophytes across four locations. A-I represents the PCoA of 16S rRNA amplicon sequencing data across location for each cultivar based on unweighted unifrac (A-C), Bray-Curtis (D-F) and Jaccard distances (G-I). A,E,H are for ND Genesis; B,E,H are for AAC Synergy while C,E,H are for Conlon genotypes.

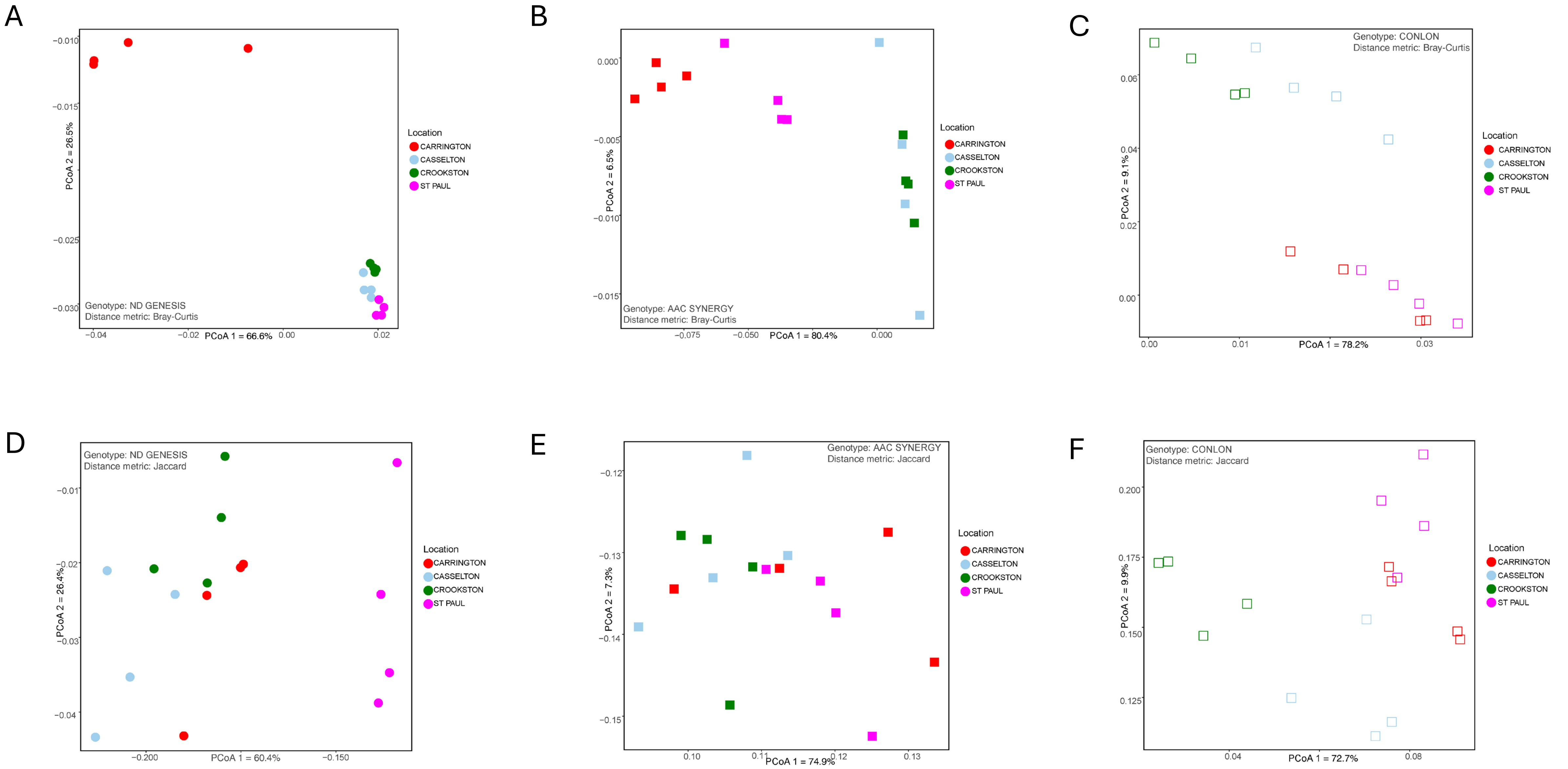

S1 Fig. S3. Fungal community composition of malting barley seed endophytes across four locations. A-F represents the PCoA of fungal ITS sequencing data across location for each cultivar based on Bray-Curtis (A-C) and Jaccard distances (D-F). A,D are for ND Genesis; B, E are for AAC Synergy and C,F are for Conlon genotypes/cultivars.

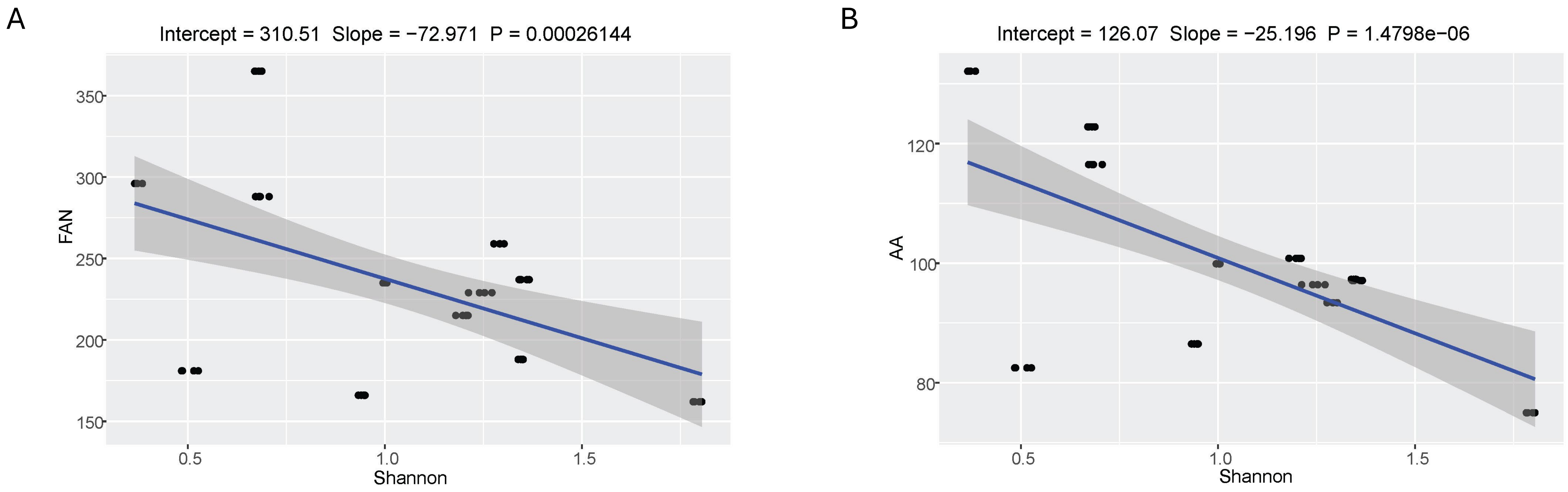

S1 Fig. S4. Negative relationship between Shannon index, bacterial genus taxa and some malt quality traits. For free amino nitrogen (FAN) (A), and alpha amylase (AA) (**B**) based on linear regression after adjusting for covariates genotype and location.

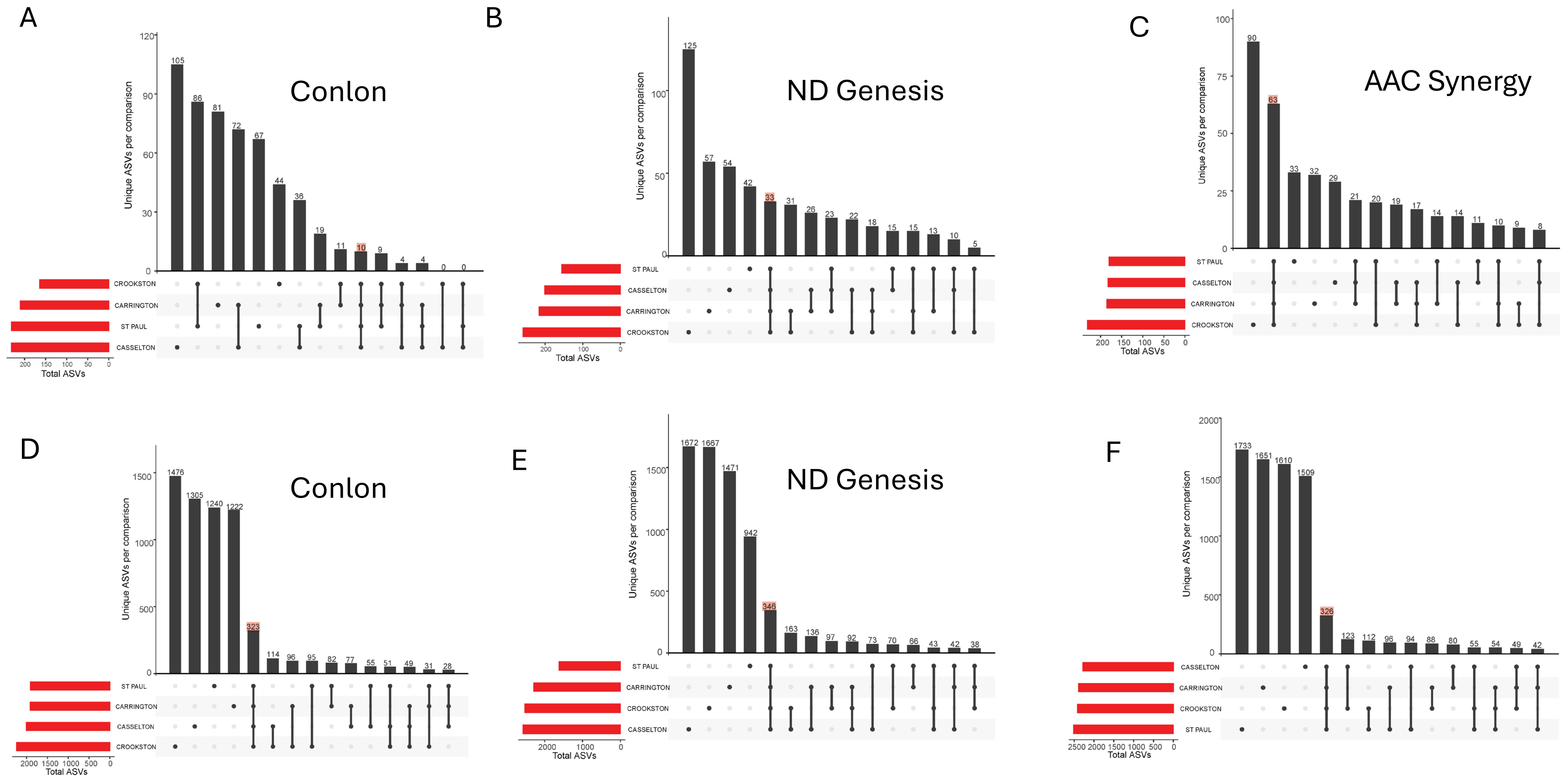

S1 Fig. S5. UpSet plots of bacterial and fungal ASVs present in each location of Conlon, ND Genesis and AAC Synergy. (A-C) UpSet plot showing the number of bacterial ASVs that are shared between or are unique for all locations for Conlon (A), ND Genesis (B) and AAC Synergy (C). (D-F) UpSet plot showing the number of fungal ASVs that are shared between or are unique for all locations for Conlon (D), ND Genesis (E) and AAC Synergy (F). Number in color in A-F indicates shared ASVs common to all locations, while filled-in black dots with an edge between the dots indicates that these ASVs are present in multiple locations.

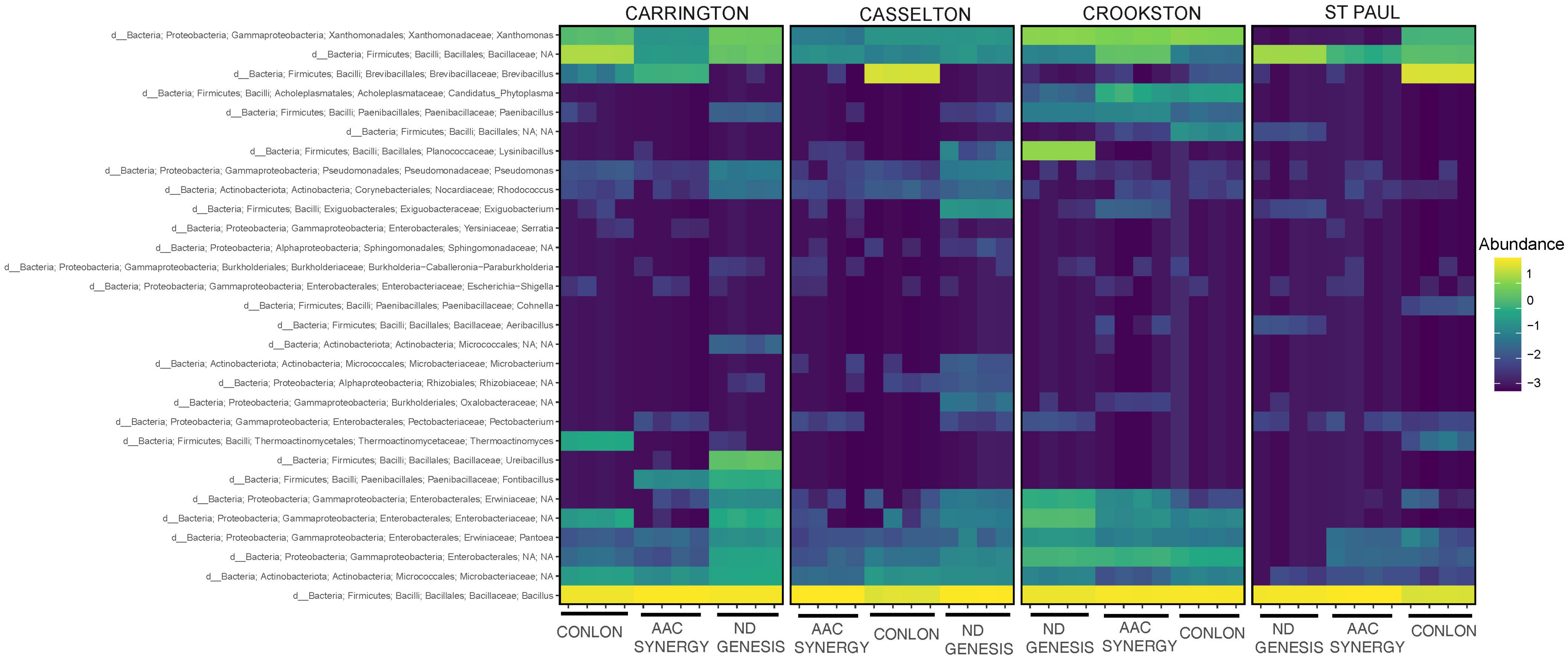

S1 Fig. S6. Heatmap of the relative abundance of the top 30 bacterial seed endophytic communities of malting barley genotypes/cultivars across four locations.

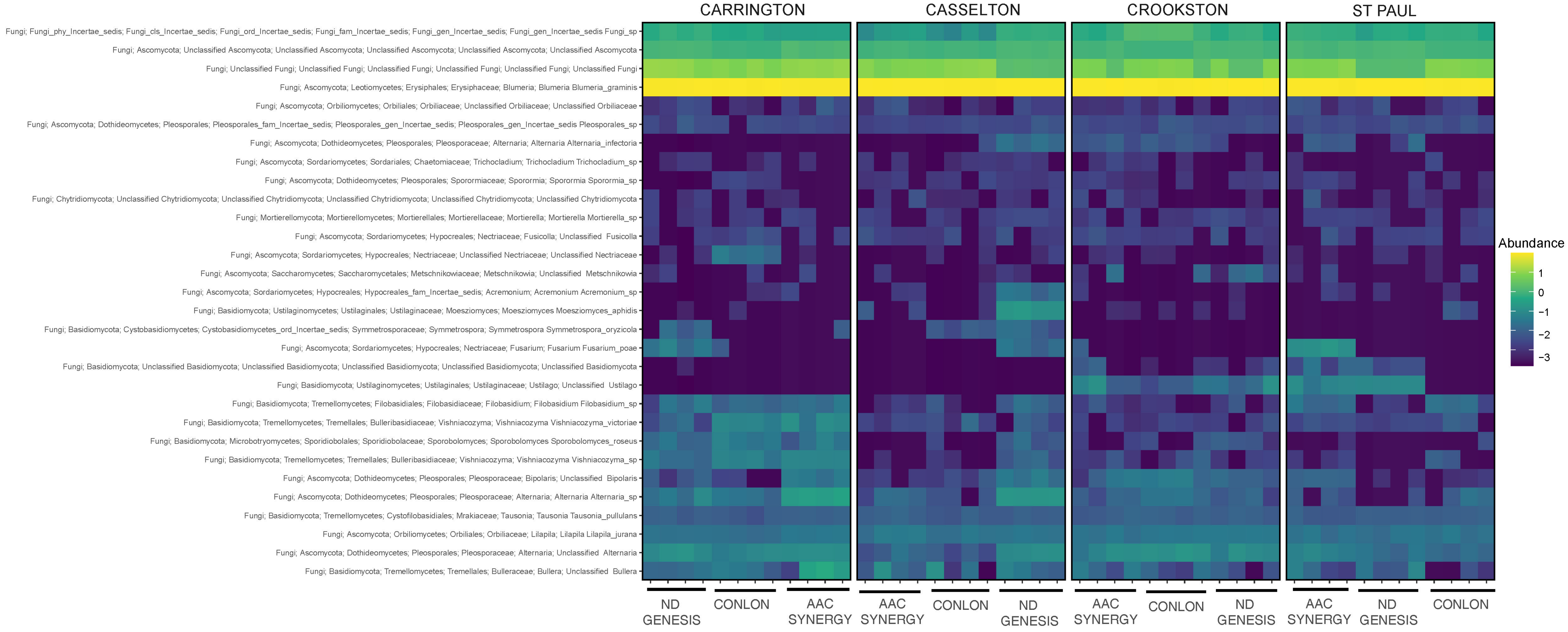

S1 Fig. S7. Heatmap of the relative abundance of the top 30 fungal seed endophytic communities of malting barley genotypes/cultivars across four locations.

A

Feature ID	Sequence Length	Sequence
7fc1d6e92ee03a814aa144f32e1f63d0	260	CCTACGGGAGGCAGCAGTAGGGAATCTTCCGCAATGGACGAAAGTCTGACGGAGCAACGCCGCGTGAGTGATGAAGGTTTTCGGATCGTAAAACTCTGTT
eead808e0197137883dc138bb21a8bdc	260	CCTACGGGAGGCAGCAGTGGGGAATTTTCCGCAATGGGCGAAAGCCTGACGGAGCAATGCCGCGTGGAGGTGGAAGGCCTACGGGTCGTCAACTTCTTTT
93c2bc2e9cbd4f85f384a1d7eb5f5377	260	CCTACGGGGGGCAGCAGTGGGGAATCTTGGACAATGGGCGAAAGCCCGATCCAGCAATATCGCGTGAGTGAAGAAGGGCAATGCCGCTTGTAAAGCTCTT
293321095e4cf6dfe46bedf510de6134	260	CCTACGGGAGGCAGCAGTAGGGAATCTTCCGCAATGGACGAAAGTCTGACGGAGCAACGCCGCGTGAGTGATGAAGGTTTTCGGATCGTAAAACTCTGTT
0810b374c63fb741fcee68eb79045c46	260	CCTACGGGAGGCAGCAGTAGGGAATCTTCCGCAATGGACGAAAGTCTGACGGAGCAACGCCGCGTGAGTGATGAAGGTTTTCGGATCGTAAAACTCTGTT
808829447d35304c65d40ab5807f4325	260	CCTACGGGAGGCAGCAGTAGGGAATCTTCCGCAATGGACGAAAGTCTGACGGAGCAACGCCGCGTGAGTGATGAAGGTTTTCGGATCGTAAAGCTCTGTT
9bc848531af1ba122a1e1e28941b935f	260	CCTACGGGTCGTCAACTTCTTTTCTCGGAGAAGAAACAATGACGGTATCTGAGGAATAAGCATCGGCTAACTCTGTGCCAGCAGCCGCGGTAAGACAGAG
e28abed63dd9831365aaced43cd1f049	260	CCTACGGGAGGCAGCAGTGGGGAATATTGGACAATGGGCGCAAGCCTGATCCAGCCATGCCGCGTGGGTGAAGAAGGCCTTCGGGTTGTAAAGCCCTTTT

Sequences producing significant alignments:

Select: All None Selected:0

Alignments Download GenBank Graphics Distance tree of results

	Description	Max Score	Total Score	Query Cover	E value	Per. Ident	Accession
☐	Xanthomonas translucens strain NRCIB_X6 16S ribosomal RNA gene, partial sequence	470	470	100%	1e-127	100.00%	gi|2148246466|OL504772.1

B

Download GenBank Graphics

Xanthomonas translucens strain NRCIB_X6 16S ribosomal RNA gene, partial sequence

Sequence ID: gi|2148246466|OL504772.1 **Length:** 1391 **Number of Matches:** 1

Range 1: 285 to 544 GenBank Graphics Next Match Previous Match

Score	Expect	Identities	Gaps	Strand
470 bits(520)	1e-127	260/260(100%)	0/260(0%)	Plus/Plus

```
Query  1    CCTACGGGAGGCAGCAGTGGGGAATATTGGACAATGGGCGCAAGCCTGATCCAGCCATGC  60
            ||||||||||||||||||||||||||||||||||||||||||||||||||||||||||||
Sbjct  285  CCTACGGGAGGCAGCAGTGGGGAATATTGGACAATGGGCGCAAGCCTGATCCAGCCATGC  344

Query  61   CGCGTGGGTGAAGAAGGCCTTCGGGTTGTAAAGCCCTTTTGTTGGGAAAGAAAAGCAGTC  120
            ||||||||||||||||||||||||||||||||||||||||||||||||||||||||||||
Sbjct  345  CGCGTGGGTGAAGAAGGCCTTCGGGTTGTAAAGCCCTTTTGTTGGGAAAGAAAAGCAGTC  404

Query  121  GGTTAATACCCGATTGTTCTGACGGTACCCAAAGAATAAGCACCGGCTAACTTCGTGCCA  180
            ||||||||||||||||||||||||||||||||||||||||||||||||||||||||||||
Sbjct  405  GGTTAATACCCGATTGTTCTGACGGTACCCAAAGAATAAGCACCGGCTAACTTCGTGCCA  464

Query  181  GCAGCCGCGGTAATACGAAGGGTGCAAGCGTTACTCGGAATTACTGGGCGTAAAGCGTGC  240
            ||||||||||||||||||||||||||||||||||||||||||||||||||||||||||||
Sbjct  465  GCAGCCGCGGTAATACGAAGGGTGCAAGCGTTACTCGGAATTACTGGGCGTAAAGCGTGC  524

Query  241  GTAGGTGGTTGTTTAAGTCC  260
            ||||||||||||||||||||
Sbjct  525  GTAGGTGGTTGTTTAAGTCC  544
```

S1 Fig. S8. Feature ID sequence identification and BlastN analysis of 16S rRNA partial sequence of Xanthomonas. (A) Feature ID colored in orange represents genus Xanthomonas (B) BlastN analysis of the 260 bp sequence shared 100% homology with Xanthomonas translucens.

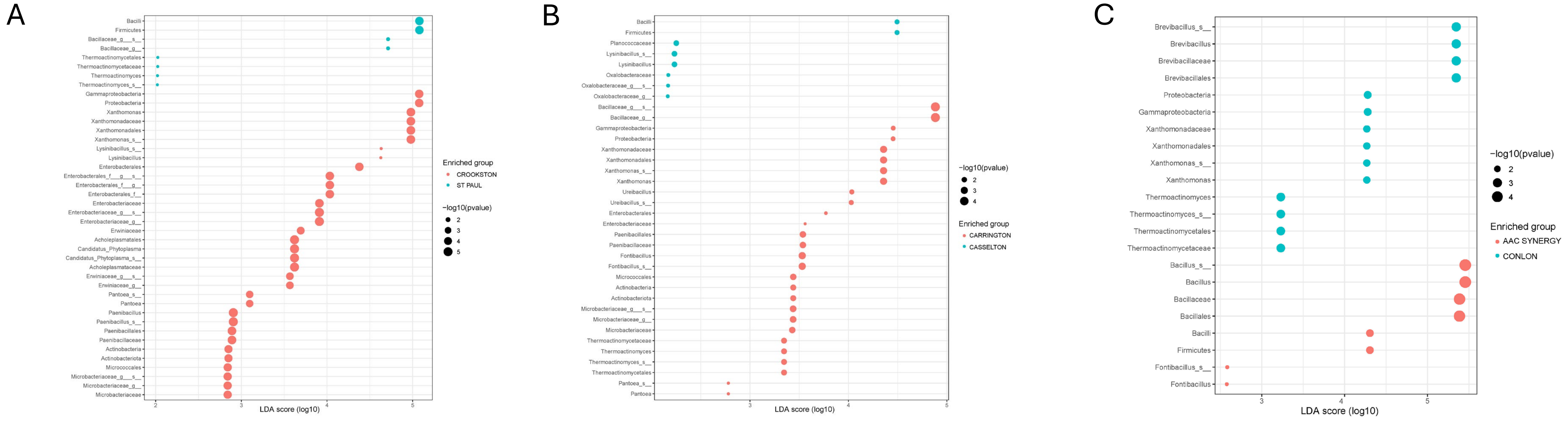

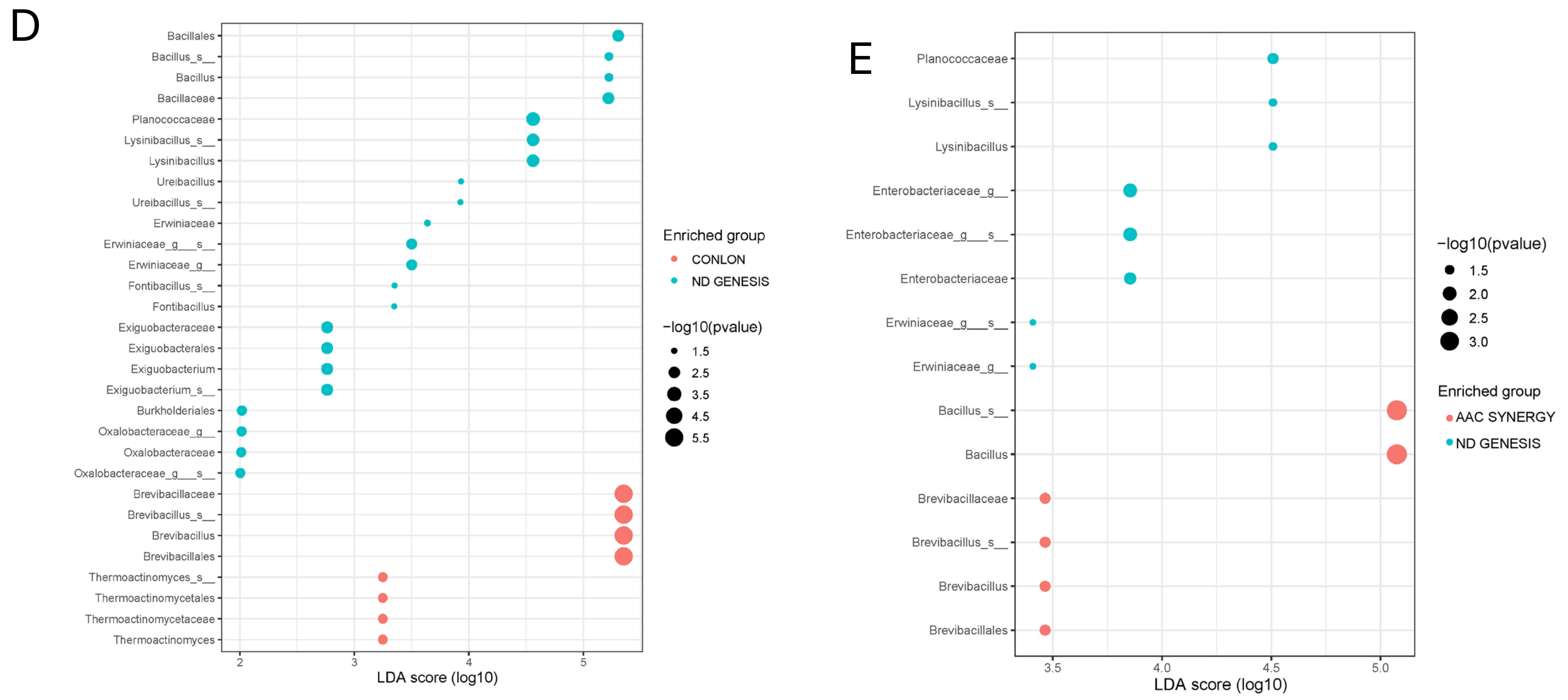

S1 Fig. S9. Genotype and location pairwise comparison of the seed endophytic bacteria microbial taxonomic composition. For Crookston vs. St. Paul (A), Carrington vs. Casselton (B), AAC Synergy vs. Conlon (C), Conlon vs. ND Genesis (D), and AAC Synergy and ND Genesis (E). For A-E, dot plots of the LDA scores (log 10) computed for features with differential abundance in each pairwise assessments as indicated above.

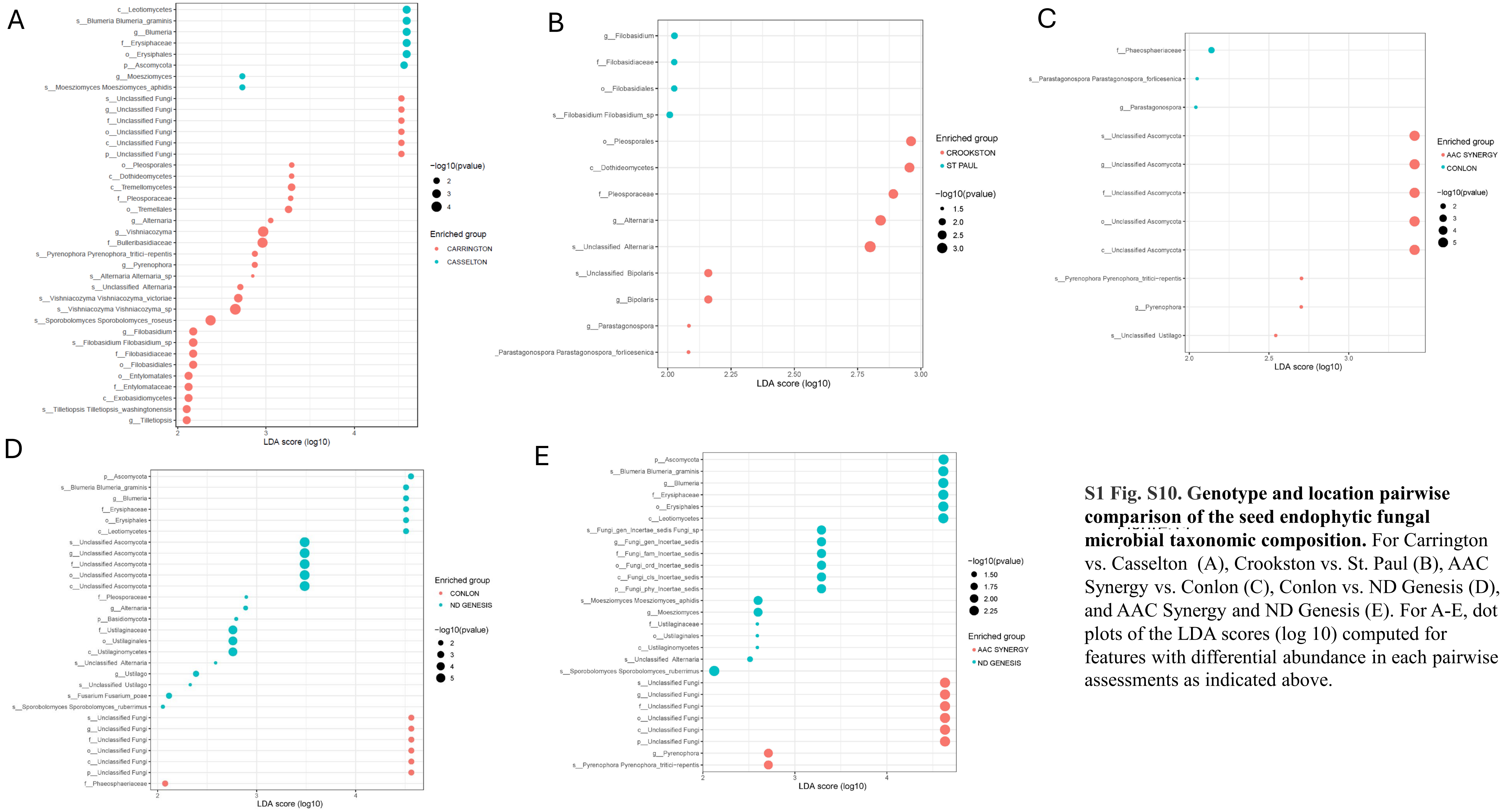

S1 Fig. S10. Genotype and location pairwise comparison of the seed endophytic fungal microbial taxonomic composition. For Carrington vs. Casselton (A), Crookston vs. St. Paul (B), AAC Synergy vs. Conlon (C), Conlon vs. ND Genesis (D), and AAC Synergy and ND Genesis (E). For A-E, dot plots of the LDA scores (log 10) computed for features with differential abundance in each pairwise assessments as indicated above.

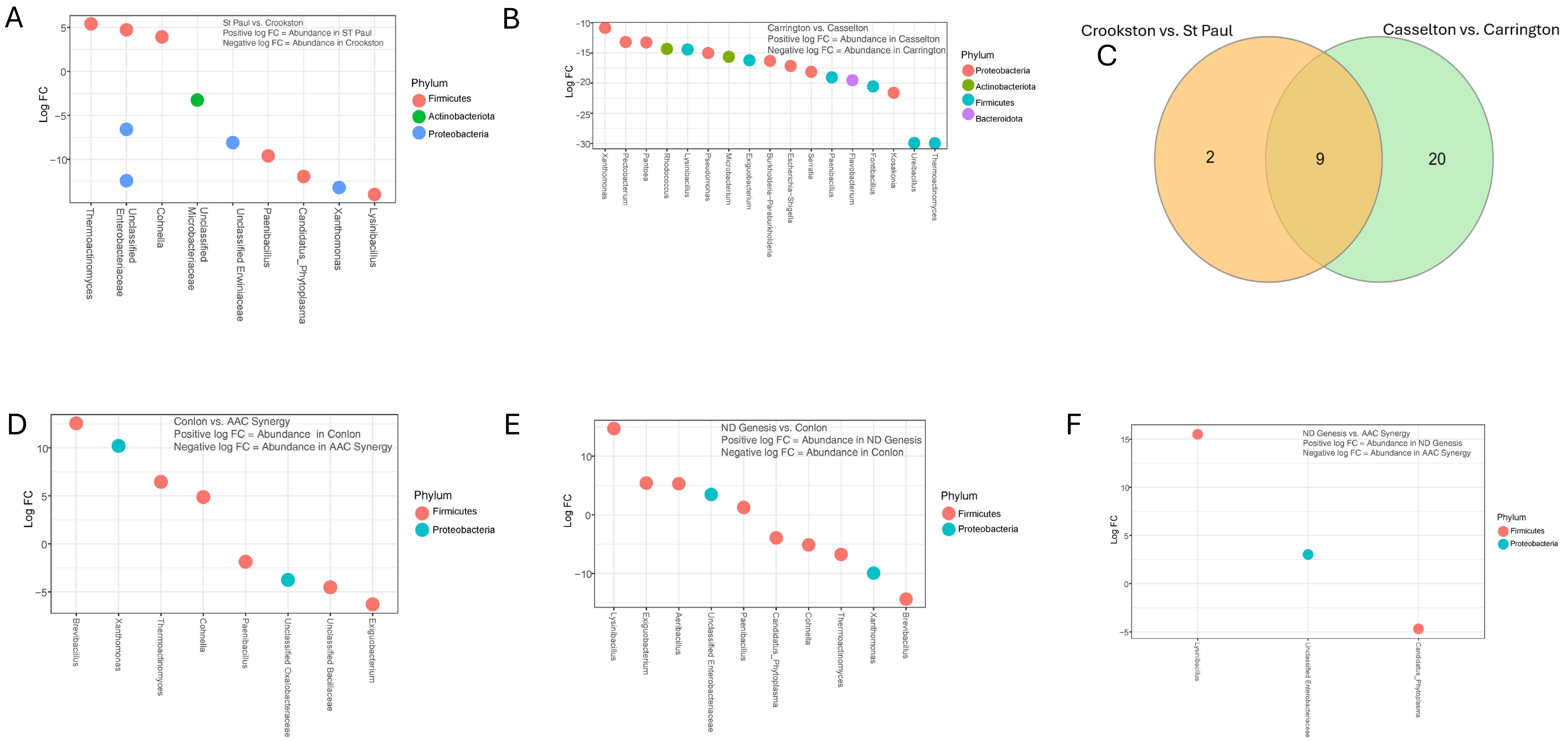
A
St Paul vs. Crookston
Positive log FC = Abundance in ST Paul
Negative log FC = Abundance in Crookston
Log FC
Phylum
Firmicutes
Actinobacteriota
Proteobacteria
Thermoactinomyces
Unclassified Enterobacteriaceae
Cohnella
Unclassified Microbacteriaceae
Unclassified Erwiniaceae
Paenibacillus
Candidatus_Phytoplasma
Xanthomonas
Lysinibacillus
B
Carrington vs. Casselton
Positive log FC = Abundance in Casselton
Negative log FC = Abundance in Carrington
Log FC
Phylum
Proteobacteria
Actinobacteriota
Firmicutes
Bacteroidota
Xanthomonas
Pectobacterium
Pantoea
Rhodococcus
Lysinibacillus
Pseudomonas
Microbacterium
Exiguobacterium
Burkholderia−Paraburkholderia
Escherichia−Shigella
Serratia
Paenibacillus
Flavobacterium
Fontibacillus
Kosakonia
Ureibacillus
Thermoactinomyces
C
Crookston vs. St Paul
Casselton vs. Carrington
2
9
20
D
Conlon vs. AAC Synergy
Positive log FC = Abundance in Conlon
Negative log FC = Abundance in AAC Synergy
Log FC
Phylum
Firmicutes
Proteobacteria
Brevibacillus
Xanthomonas
Thermoactinomyces
Cohnella
Paenibacillus
Unclassified Oxalobacteraceae
Unclassified Bacillaceae
Exiguobacterium
E
ND Genesis vs. Conlon
Positive log FC = Abundance in ND Genesis
Negative log FC = Abundance in Conlon
Log FC
Phylum
Firmicutes
Proteobacteria
Lysinibacillus
Exiguobacterium
Aeribacillus
Unclassified Enterobacteriaceae
Paenibacillus
Candidatus_Phytoplasma
Cohnella
Thermoactinomyces
Xanthomonas
Brevibacillus
F
ND Genesis vs. AAC Synergy
Positive log FC = Abundance in ND Genesis
Negative log FC = Abundance in AAC Synergy
Log FC
Phylum
Firmicutes
Proteobacteria
Lysinibacillus
Unclassified Enterobacteriaceae
Candidatus_Phytoplasma

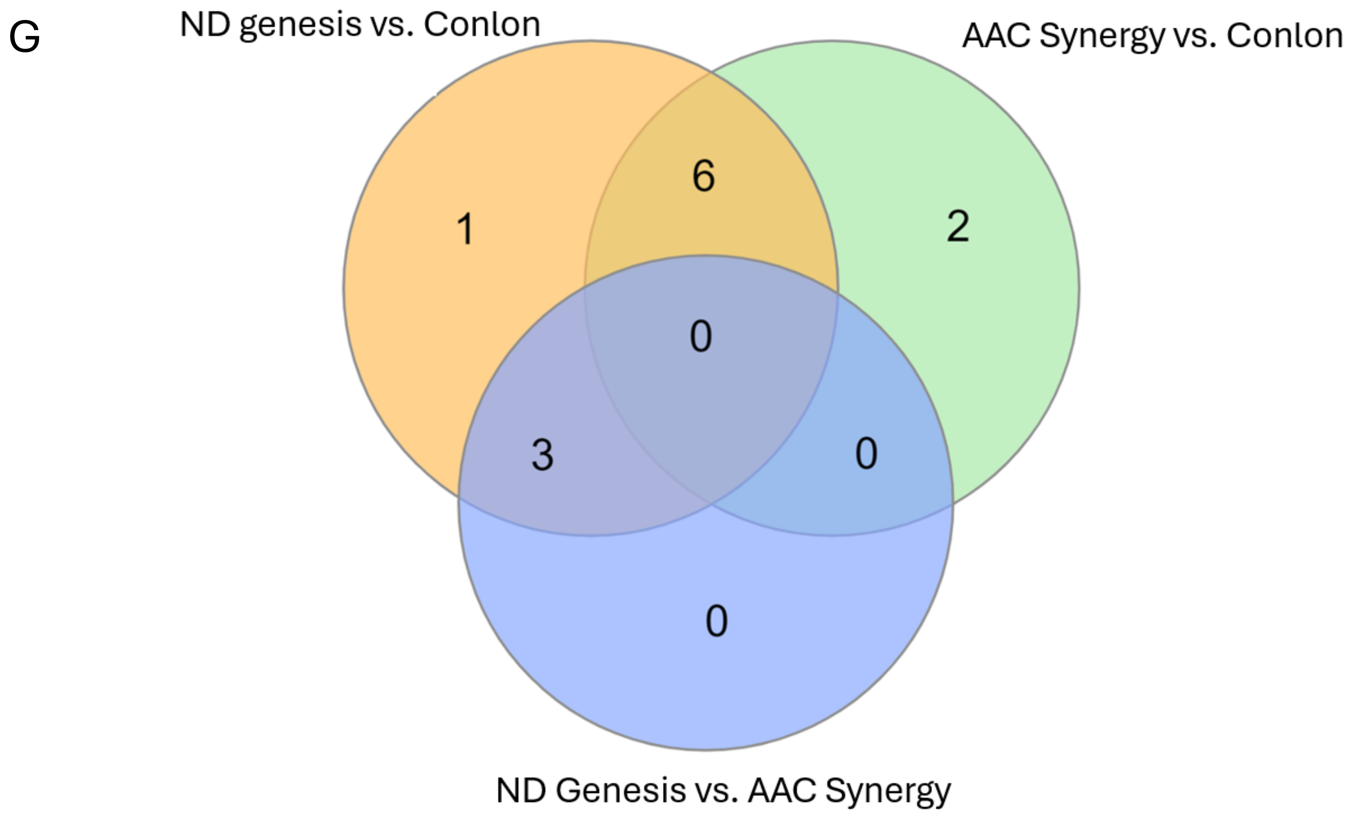

S1 Fig. S11. Differential abundance analysis of seed endophytic bacterial communities of malting barley grown across four locations. (**A**) Pairwise differential abundance analysis (based on logFC) between St. Paul and Crookston locations at the genus level. Positive logFC indicates increased endophytic bacterial (EB) abundance in St Paul (decrease EB in Crookston), while negative logFC indicates decreased EB in St. Paul (increased EB in Crookston). (**B**) Pairwise differential abundance analysis (based on logFC) between Carrington and Casselton locations at the genus level. The negative logFC indicates decreased EB in Casselton (increased EB in Carrington). Only some genera are reported (for complete list see Table S7) (**C**). Venn diagram showing unique and shared ASVs between pairwise location comparisons (Complete list is available in Table S7). (**D -F**) Pairwise differential abundance analysis (based on logFC) between Conlon and AAC Synergy (**D**), Conlon and ND Genesis (**E**) and AAC Synergy and ND Genesis (**F**) at the genus level. For **D**, Positive logFC indicates increased EB abundance in Conlon (decrease EB in AAC Synergy), while negative logFC indicates decreased EB in Conlon (increased EB in AAC Synergy). For **E**, Positive logFC indicates increased EB abundance in Conlon (decrease EB in ND Genesis), while negative logFC indicates decreased EB in Conlon (increased EB in ND Genesis). For **F**, Positive logFC indicates increased EB abundance in AAC Synergy (decrease EB in ND Genesis), while negative logFC indicates decreased EB in AAC Synergy (increased EB in ND Genesis). (**G**) Venn diagram showing unique and shared ASVs between pairwise genotype comparisons (Complete list is available in Table S7).

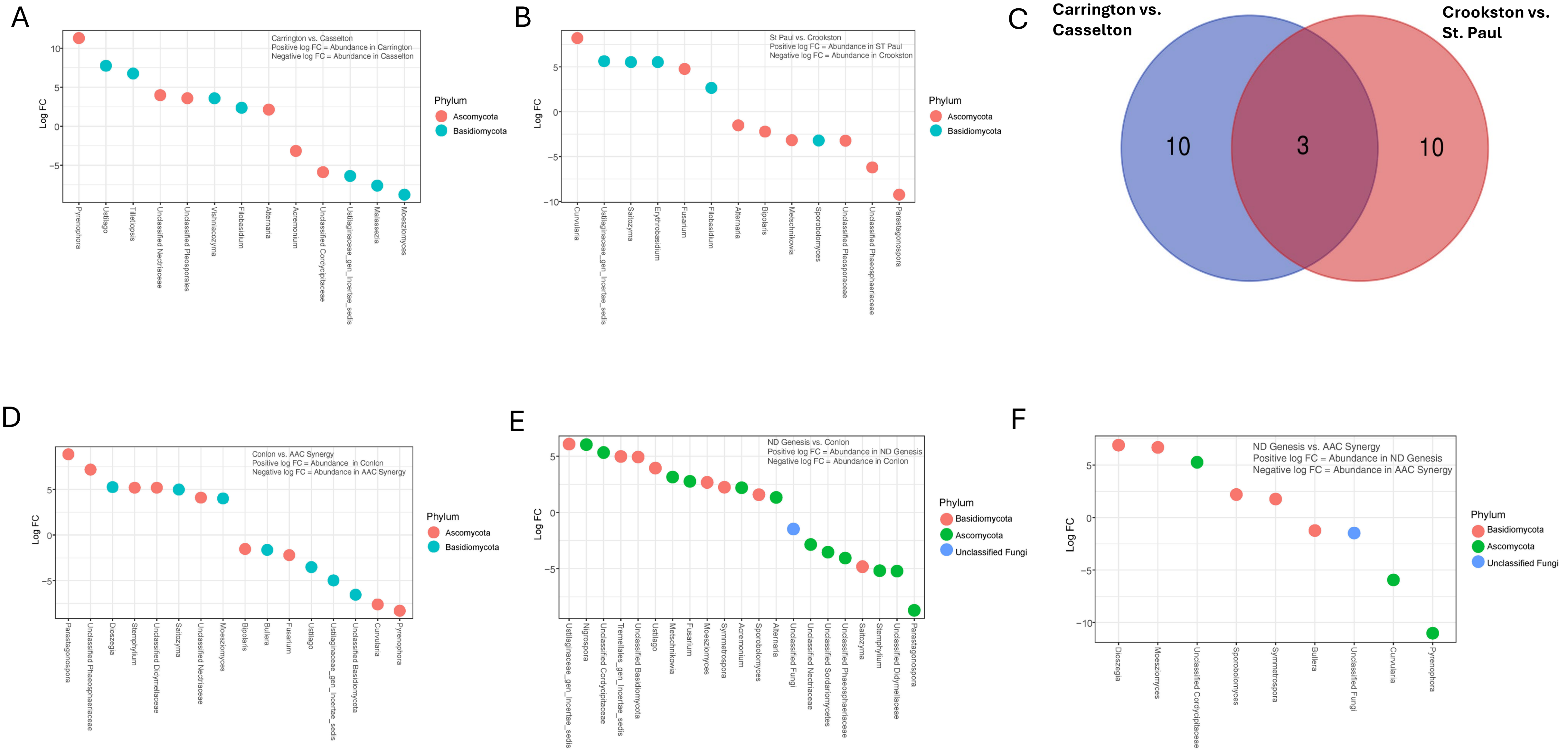
A
Carrington vs. Casselton
Positive log FC = Abundance in Carrington
Negative log FC = Abundance in Casselton
Log FC
Phylum
Ascomycota
Basidiomycota
Pyrenophora
Ustilago
Tilletopsis
Unclassified Nectriaceae
Unclassified Pleosporales
Vishniacozyma
Filobasidium
Alternaria
Acremonium
Unclassified Cordycipitaceae
Ustilaginaceae_gen_Incertae_sedis
Malassezia
Moesziomyces
B
St Paul vs. Crookston
Positive log FC = Abundance in ST Paul
Negative log FC = Abundance in Crookston
Log FC
Phylum
Ascomycota
Basidiomycota
Curvularia
Ustilaginaceae_gen_Incertae_sedis
Saitozyma
Erythrobasidium
Fusarium
Filobasidium
Alternaria
Bipolaris
Metschnikowia
Sporobolomyces
Unclassified Pleosporaceae
Unclassified Phaeosphaeriaceae
Parastagonospora
C
Carrington vs. Casselton
Crookston vs. St. Paul
10
3
10
D
Conlon vs. AAC Synergy
Positive log FC = Abundance in Conlon
Negative log FC = Abundance in AAC Synergy
Log FC
Phylum
Ascomycota
Basidiomycota
Parastagonospora
Unclassified Phaeosphaeriaceae
Dioszegia
Stemphylium
Unclassified Didymellaceae
Saitozyma
Unclassified Nectriaceae
Moesziomyces
Bipolaris
Bullera
Fusarium
Ustilago
Ustilaginaceae_gen_Incertae_sedis
Unclassified Basidiomycota
Curvularia
Pyrenophora
E
ND Genesis vs. Conlon
Positive log FC = Abundance in ND Genesis
Negative log FC = Abundance in Conlon
Log FC
Phylum
Basidiomycota
Ascomycota
Unclassified Fungi
Ustilaginaceae_gen_Incertae_sedis
Nigrospora
Unclassified Cordycipitaceae
Tremellales_gen_Incertae_sedis
Unclassified Basidiomycota
Ustilago
Metschnikowia
Fusarium
Moesziomyces
Symmetrospora
Acremonium
Sporobolomyces
Alternaria
Unclassified Fungi
Unclassified Nectriaceae
Unclassified Sordariomycetes
Unclassified Phaeosphaeriaceae
Saitozyma
Stemphylium
Unclassified Didymellaceae
Parastagonospora
F
ND Genesis vs. AAC Synergy
Positive log FC = Abundance in ND Genesis
Negative log FC = Abundance in AAC Synergy
Log FC
Phylum
Basidiomycota
Ascomycota
Unclassified Fungi
Dioszegia
Moesziomyces
Unclassified Cordycipitaceae
Sporobolomyces
Symmetrospora
Bullera
Unclassified Fungi
Curvularia
Pyrenophora

G

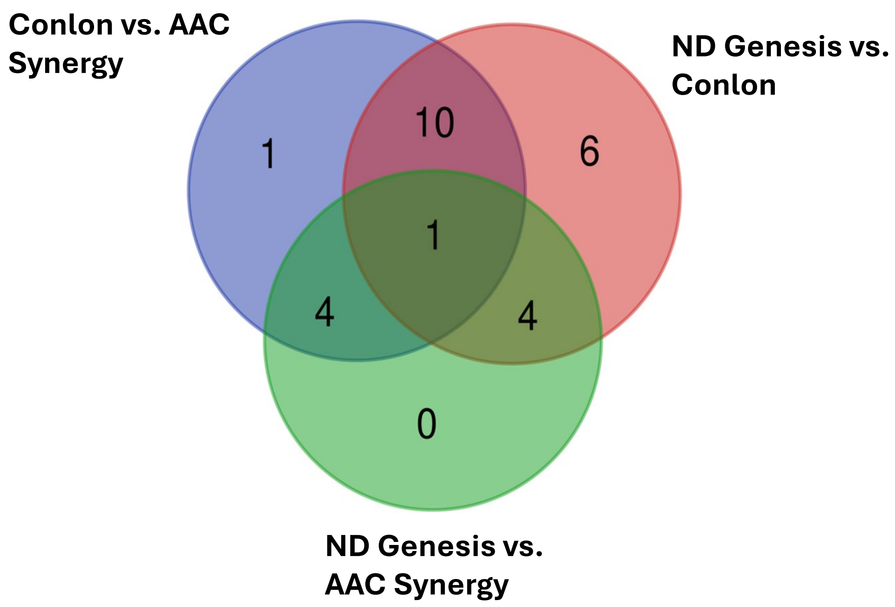

S1 Fig. S12. Differential abundance analysis of seed endophytic fungal communities of malting barley grown across four locations. (**A**) Pairwise differential abundance analysis (based on logFC) between Carrington and Casselton locations at the genus level. Positive logFC indicates increased endophytic bacterial (EB) abundance in Carrington (decrease EB in Casselton), while negative logFC indicates decreased EB in Carrington (increased EB in Casselton). (**B**) Pairwise differential abundance analysis (based on logFC) between St. Paul and Crookston locations at the genus level. Positive logFC indicates increased endophytic bacterial (EB) abundance in St. Paul (decrease EB in Crookston), while negative logFC indicates decreased EB in St. Paul (increased EB in Crookston). (**C**) Venn diagram showing unique and shared ASVs between pairwise location comparisons (Complete list is available in Table S8). (**D -F**) Pairwise differential abundance analysis (based on logFC) between Conlon and AAC Synergy (**D**), ND Genesis and Conlon (**E**) and ND Genesis and AAC Synergy (**F**) at the genus level. For **D**, Positive logFC indicates increased EB abundance in Conlon (decrease EB in AAC Synergy), while negative logFC indicates decreased EB in Conlon (increased EB in AAC Synergy). For **E**, Positive logFC indicates increased EB abundance in ND Genesis (decrease EB in Conlon), while negative logFC indicates decreased EB in ND Genesis (increased EB in Conlon). For **F**, Positive logFC indicates increased EB abundance in ND Genesis (decrease EB in AAC Synergy), while negative logFC indicates decreased EB in ND Genesis (increased EB in AAC Synergy). (**G**) Venn diagram showing unique and shared ASVs between pairwise genotypes comparisons (Complete list is available in Table S8).
